# Reconciling structure prediction of alloyed, ultrathin nanowires with spectroscopy†

**DOI:** 10.1039/d1sc00627d

**Published:** 2021-04-26

**Authors:** Scott C. McGuire, Amani M. Ebrahim, Nathaniel Hurley, Lihua Zhang, Anatoly I. Frenkel, Stanislaus S. Wong

**Affiliations:** Department of Chemistry, Stony Brook University Stony Brook New York 11794-3400 USA stanislaus.wong@stonybrook.edu; Department of Materials Science and Chemical Engineering, Stony Brook University Stony Brook New York 11794-2275 USA anatoly.frenkel@stonybrook.edu; Center for Functional Nanomaterials, Brookhaven National Laboratory Upton New York 11973 USA; Chemistry Division, Brookhaven National Laboratory Upton New York 11973 USA

## Abstract

A number of complementary, synergistic advances are reported herein. First, we describe the ‘first-time’ synthesis of ultrathin Ru_2_Co_1_ nanowires (NWs) possessing average diameters of 2.3 ± 0.5 nm using a modified surfactant-mediated protocol. Second, we utilize a combination of quantitative EDS, EDS mapping (along with accompanying line-scan profiles), and EXAFS spectroscopy results to probe the local atomic structure of not only novel Ru_2_Co_1_ NWs but also ‘control’ samples of analogous ultrathin Ru_1_Pt_1_, Au_1_Ag_1_, Pd_1_Pt_1_, and Pd_1_Pt_9_ NWs. We demonstrate that ultrathin NWs possess an atomic-level geometry that is fundamentally dependent upon their intrinsic chemical composition. In the case of the PdPt NW series, EDS mapping data are consistent with the formation of a homogeneous alloy, a finding further corroborated by EXAFS analysis. By contrast, EXAFS analysis results for both Ru_1_Pt_1_ and Ru_2_Co_1_ imply the generation of homophilic structures in which there is a strong tendency for the clustering of ‘like’ atoms; associated EDS results for Ru_1_Pt_1_ convey the same conclusion, namely the production of a heterogeneous structure. Conversely, EDS mapping data for Ru_2_Co_1_ suggests a uniform distribution of both elements. In the singular case of Au_1_Ag_1_, EDS mapping results are suggestive of a homogeneous alloy, whereas EXAFS analysis pointed to Ag segregation at the surface and an Au-rich core, within the context of a core–shell structure. These cumulative outcomes indicate that only a combined consideration of both EDS and EXAFS results can provide for an accurate representation of the local atomic structure of ultrathin NW motifs.

## Abstract 2

EDS and EXAFS spectroscopy are used as complementary techniques to investigate the local structure of bimetallic ultrathin nanowires. Results highlight the importance of using a combined approach to achieve an accurate understanding of these systems.

## Graphical abstract

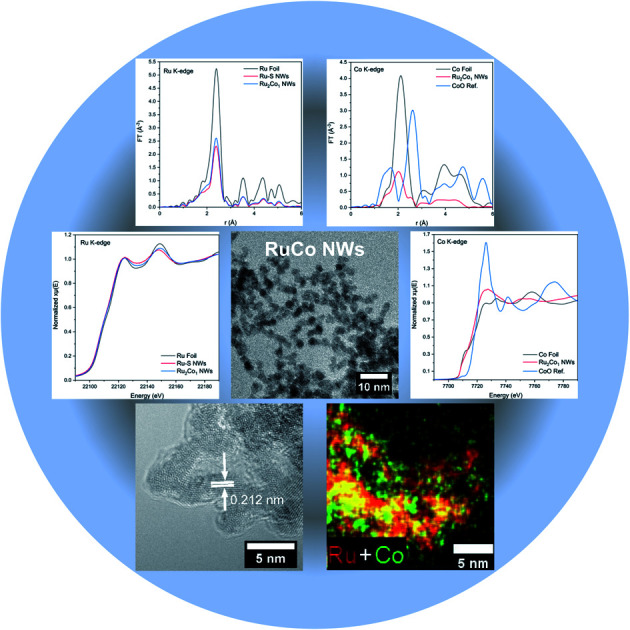

## 1. Introduction

Recent literature has demonstrated how tunable parameters such as (i) size, (ii) morphology, (iii) chemical composition, and (iv) atomic structure can impact upon the performance of functional electrocatalysts.^1–3^ Specifically, nanoscale formulations of catalysts allow for a higher surface area-to-volume ratio and reduce the overall metal loading, which can thereby lower expected costs. Moreover, as compared with conventional zero-dimensional (0D) nanoparticles (NPs), anisotropic one-dimensional (1D) nanowires (NWs) are less susceptible to dissolution, Ostwald ripening, and aggregation, which promote enhanced stability and durability. Furthermore, NWs not only evince enhanced electron and mass transport but also enable the exposure of specific crystalline planes which can be beneficial for catalytic activity. In particular, ultrathin NWs represent an advantageous architectural target in that they are expected to maintain slightly contracted surfaces, which can weaken the interaction with surface passivating O_2_. Our previous studies^4–13^ in this area also suggest that ultrathin NWs are generally chemically homogeneous and structurally monodisperse, while maintaining fewer defect sites.

As implied earlier, the chemical composition of the electrocatalysts also plays a key role in dictating performance. Whereas Pt is indeed the most active metal as a catalyst for a number of small-molecule reactions such as the methanol oxidation reaction (MOR), Pt is also relatively scarce and expensive. Significantly, Pt is prone to slow reaction kinetics due to its propensity to adsorb CO, which poisons the surface by limiting the available active sites. The CO tolerance of Pt-based catalysts can be improved upon by the introduction of additional metals to promote the formation of OH species on the surface, which can subsequently react with and oxidize the poisoning CO.^14^ In particular, theory and experiment have shown that Ru is highly effective in increasing CO tolerance and thereby improving reaction kinetics.^15^ In addition, the lattice mismatch between Pt and Ru introduces strain which can decrease the binding energy of the reaction intermediates, thereby further increasing CO tolerance.^16^ Similar types of behavior have been observed upon the alloying of Pt with non-noble metals, such as Cu, Fe, Sn, Pb or Zn.^17–22^ In the ESI,† we discuss in much greater detail the broader implications of morphology and chemical composition upon electrocatalytic activity.

Nevertheless, relatively little work has been performed on RuCo alloys, which have been reported to be useful in applications, such as but not limited to batteries^23^ and Fischer–Tropsch catalysts^24,25^ in addition to electrocatalysts for the hydrogen evolution reaction (HER),^22,26–30^ the hydrogen oxidation reaction (HOR),^31^ the oxygen reduction reaction (ORR),^32,33^ and the oxygen evolution reaction (OER).^34^ Results for RuCo alloys used as electrocatalysts have been especially promising, with the alloys frequently outperforming the Pt standards at lower overall costs. Specifically, a study which compared RuM (M = Co, Ni, Fe) catalysts for the HOR, HER, ORR, and OER reported that the RuM alloys achieved higher activities for HOR and HER *versus* both pure Ru and Pt standards.^31^ In addition, it was determined that the RuCo catalysts yielded the highest activity for HOR, HER, and OER, out of all of the RuM alloys tested.

Nonetheless, fewer studies have been carried out on the synthesis and characterization of RuCo alloys, possessing different morphologies. In effect, the majority of prior literature on RuCo has centered on 0D NPs with a smaller number of reports on the synthesis of discrete morphologies, such as 1D NW arrays^35^ and two-dimensional (2D) nanosheets.^27^ As such, herein, we have focused on developing a novel and potentially generalizable method for the production of ultrathin RuCo NWs. Our customized protocol employs oleylamine (OAm) and oleic acid (OAc) with the former in the role of a surfactant and reducing agent and the latter in the capacity of an additional surfactant, with which to collectively guide the growth of 1D nanostructures.

We have characterized the composition, crystallinity, morphology, and local atomic structure of our as synthesized Ru_2_Co_1_ NWs with a combination of various techniques, including X-ray diffraction (XRD), transmission electron microscopy (TEM), high resolution TEM (HRTEM), energy dispersive X-ray spectroscopy (EDS), and X-ray absorption spectroscopy (XAS). Significantly herein, we probe the structural results provided by EDS *versus* XAS, the latter of which arguably enables a more accurate determination of the local atomic structure through an analysis of the extended X-ray absorption fine structure (EXAFS) region.

The EXAFS signal incorporates information about coordination numbers, interatomic distances, and the nature of disorder within systems (due to both static and dynamic displacements of all atoms from their average positions).^36^ In particular, the EXAFS spectra yield information about the number, type of, and distance to the atoms surrounding the central, X-ray absorbing atom. As such, the coordination numbers (*N*) of atomic pairs within bimetallic alloyed NPs are often used to differentiate between different types of short-range order in these NPs, and/or ascertain the degree of compositional monodispersity within the sample. Indeed, EXAFS analysis can be used to confirm alloy formation and to distinguish between different alloying motifs (*e.g.*, random or core–shell-like non-random).^37,38^ For instance, by analyzing *N* values associated with the bonds within Pd–Au NPs, it was concluded that Pd atoms predominantly resided on the surface of these NPs, whereas Au atoms primarily localized in the core.^37^ Nevertheless, one of the continuing challenges with respect to the precise, atomistic characterization of the nanocatalyst geometry, especially anisotropic, 1D NW motifs, is the relative difficulty in terms of ascertaining their nuanced geometric and compositional structure with precise and useful spatial resolution.^36^

What factors can complicate the analysis? First, it has been suggested^39^ that in a Pt–Ru alloy, if there are two populations of Pt–Ru bonds at an interface as an example, the strongly ordered and strongly disordered ones, the EXAFS signal will be dominated by the relatively ordered pairs (*i.e.*, ones with smaller disorder parameters), whereas the second population of bonds, the strongly disordered ones, will give a much weaker contribution to EXAFS. Second, even the relationship between the alloying tendency and chemical composition within nanomaterials can be complex. For example, density functional theory (DFT) calculations have shown that Pd evinces a strong surface segregation preference when acting as an atom impurity within a Fe host, whereas a Fe impurity atom within a Pd host shows a greater propensity for strong anti-segregation.^40^ Third, the synthetic protocol makes a difference. With commercial Pd–Pt bimetallic NPs, EXAFS results were consistent with a structure in which Pt atoms are enriched in the core and Pd atoms are localized in the shell, with a greater extent of Pd atomic dispersion in the NPs.^41^ By contrast, with PdPt catalysts dispersed in zeolites, XAS analysis suggested that Pd and Pt were present mainly as the corresponding oxide NPs and aggregates.^42^ Fourth, when the distribution of bonds is strongly asymmetric, emanating from factors such as but not limited to surface tension, which are particularly important in influencing the behavior of nanoparticles with characteristic sizes of less than *ca.* 5 nm, the results of conventional EXAFS fitting exhibited significant artifacts, such as an under-estimation of the coordination number and bond length disorder.^43–45^ Fifth, the precise morphology itself is relevant. For example, the average *N* value of Pt within so-called ‘excavated’ nanoframes (that had been subjected to an extra processing step) was found to be higher than that of their hollow nanoframe counterparts.^46^

All of these different parameters were clearly significant in interpreting a study^47^ of the structural evolution of mesostructured PtRu NPs generated within a lyotropic liquid-crystalline template. The X-ray absorption near edge structure (XANES) data at the Ru L_2,3_– and Pt L_3_– edges highlighted predominantly metallic states of Ru and Pt within the PtRu NPs upon electroreduction. Nevertheless, a more rapid reduction of Pt precursors coupled with a release of Ru atoms from Ru precursors in two steps upon electroreduction resulted in aggregation into PtRu NPs, consisting of a Pt-rich core, a Ru-rich shell, and a greater extent of Ru segregation.

Therefore, our novel contribution to the literature herein has been to apply EXAFS to investigate the structure and dispersion of elements within bimetallic ultrathin Ru_2_Co_1_ NWs. Moreover, as comparative systems, we have also synthesized and characterized not only various other bimetallic alloyed NWs with distinctive compositions of Ru_1_Pt_1_, Au_1_Ag_1_, Pd_1_Pt_1_, and Pd_1_Pt_9_ but also monometallic Ru, Pt, Pd, and Au NWs as controls. All of these samples serve as a means of simultaneously systematically and quantitatively comparing and contrasting the structures, determined by EXAFS analysis, with which to achieve insights into local atomic structure, *versus* data obtained using more frequently utilized techniques such as standard EDS. An important motivation for our current project has been in correlating TEM EDS mapping with EXAFS analysis to analyze and compare the findings of each measurement mode.

What makes our work even more impactful is that there are only a few reports exploring either the ‘short range order parameter’^36,48^ or a conceptually analogous method developed by Huang *et al.* with which to investigate and quantitatively assess the structure of nanoscale morphologies, such as nanoframes,^46,49^ nanorods,^50^ and nanochains^51^ at the atomic level using EXAFS. In this study, to probe the implications and applicability of these prior theoretical studies, we have analyzed the structures of our range of bimetallic ultrathin NWs using the short range order parameter, which is essentially a structure-dependent function of *N*. We have also highlighted the limits of applicability of this parameter; specifically, while effective as a useful indicator of either positive or negative tendency to clustering of ‘like’ atoms, it is fundamentally limited to the analysis of relatively homogeneous alloys only.

Our combined results indicate that the Ru-based NWs (*i.e.*, Ru_2_Co_1_ and Ru_1_Pt_1_) both form as homophilic structures in which there is a strong tendency for clustering of ‘like’ atoms. By comparison, the Au_1_Ag_1_ NWs likely are synthesized as a core–shell motif, incorporating an Au-rich core with an Ag-rich shell. By contrast, the Pd_1_Pt_9_ NWs are generated as a homogeneous alloy structure, whereas the Pd_1_Pt_1_ are produced as an alloy structure with a slight tendency towards the clustering of ‘like’ atoms. Therefore, to highlight the key points of novelty, we not only report herein for the first time on the synthesis of uniform, ultrathin Ru_2_Co_1_ NWs but also for the first time, apply quantitative EXAFS analysis techniques towards the investigation of the local atomic structure within various ultrathin bimetallic NW systems.

## 2. Results and discussion

### Synthesis and characterization

#### Ru-based NWs

To emphasize a key contribution in our article, we have reported on the first viable synthesis of ultrathin RuCo NWs. The precise combination of composition and morphology, to the best of our knowledge, had not been previously produced before by any reported protocol. Our methodology was inspired by reports, which have utilized oleylamine as both a surfactant and a reducing agent in the formation of not only PtM (M = Co, Ni, Zn, Cu, Fe) but also Ru NPs. The key discovery was that using a combination of oleylamine and oleic acid resulted in the formation of ultrathin NWs.

As initial work, experiments had been performed for producing pure Ru, wherein solely oleylamine (OAm) was employed in the individual roles of reaction medium, surfactant, and reducing agent, simultaneously. This procedure resulted in the formation of Ru NPs with average diameters of 3.7 ± 0.5 nm, as shown in Fig. S1A.† Upon the addition of oleic acid (OAc) as an extra surfactant, we isolated ultrathin NWs with average measured diameters of 2.6 ± 0.5 nm, as shown in Fig. S1B.† As such, herein, OAm acts as both a reducing agent and a surfactant, whereas OAc behaves as a supplementary surfactant agent. In terms of a specific chemical role in the reaction, it has been previously proposed that both OAm and OAc can behave as capping ligands to effectively guide and enable the formation of NWs through a process of oriented attachment.^60–63^

**Fig. 1:**
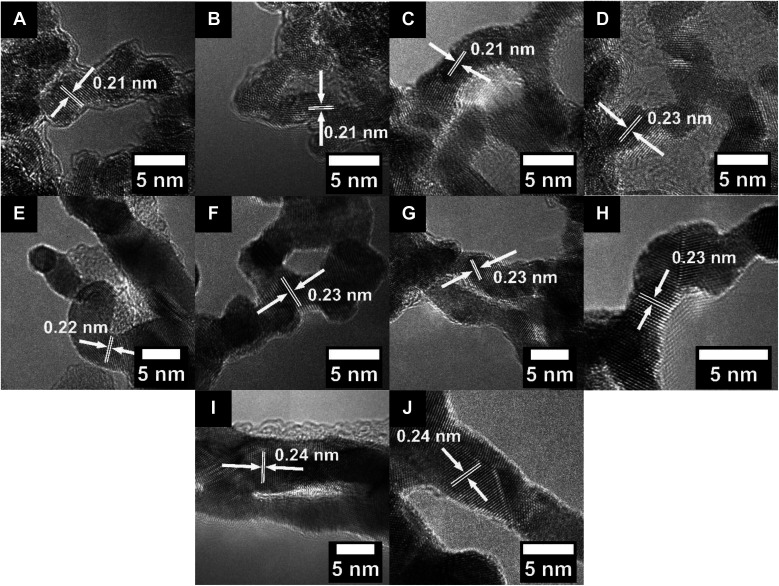
HRTEM images with the measured *d*-spacing values for (A) Ru–S, (B) Ru_2_Co_1_, (C) Ru–H, (D) Ru_1_Pt_1_, (E) Pd, (F) Pt, (G) Pd_1_Pt_1_, (H) Pd_1_Pt_9_, (I) Au, and (J) Au_1_Ag_1_ NWs, respectively.

This procedure was successful in synthesizing Ru and RuCo NWs (referred to herein as Ru–S and Ru_2_Co_1_, respectively, which more accurately designate the synthetic protocol used and nominal concentrations associated with these materials) with average diameters of 2.6 ± 0.5 and 2.3 ± 0.5 nm, respectively, as determined by TEM (Fig. S2A and B†). Moreover, the *d*-spacings of the Ru–S and Ru_2_Co_1_ NWs were both measured to be 0.21 nm, as derived from the associated HRTEM images (Fig. 1A and B); these lattice parameter readings could be assigned to the (101) plane of *hcp* Ru.^64^

**Fig. 2:**
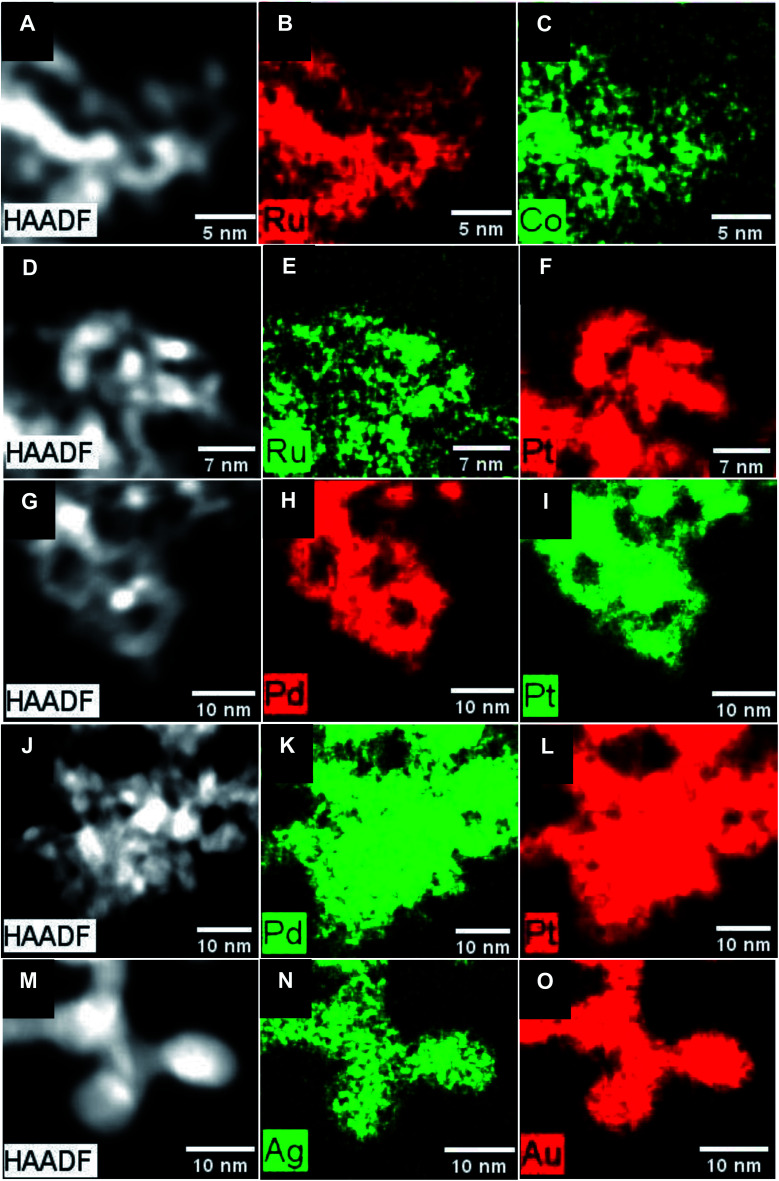
(A, D, G, J and M) HAADF-STEM images and (B, C, E, F, H, I, K, L, N and O) the corresponding EDS mapping data associated with (A–C) Ru_2_Co_1_, (D–F) Ru_1_Pt_1_, (G–I) Pd_1_Pt_1_, (J–L) Pd_1_Pt_9_, and (M–O) Au_1_Ag_1_ NWs, respectively.

The chemical composition and crystallinity of the as-prepared NWs were further characterized by XRD (Fig. S3A†); both systems exhibit XRD patterns associated with *hcp* Ru. Hence, the lack of any apparent impurity peaks was suggestive of the acceptable purity of our samples. In terms of the actual isolated stoichiometry, it is worth noting that with the Ru_2_Co_1_ NWs, whereas an initial precursor Ru : Co molar feed ratio of 2 : 1 was used for their generation, EDS measurements implied that the definitive amount of elemental incorporation was different. Specifically, as-prepared samples integrated a measured Ru : Co content in the molar ratio of 82 : 18, with elemental mapping (Fig. 2B and C) data indicating that Ru and Co appear to be evenly and uniformly distributed throughout the NWs. Moreover, the associated EDS line-scan profile (Fig. S4A†) further supports the idea of a relatively homogeneous distribution of these elements within the NWs. For experiments wherein the amount of Co precursor was increased to a Ru : Co molar ratio of 1 : 2 and beyond, the resulting XRD patterns were indicative of the formation of cobalt carbide (Fig. S5†). This finding suggested that the maximum amount of Co that could be incorporated within the NWs to enable viable alloy formation was ∼20%.

As ‘controls’, RuPt and Ru ultrathin NWs (denoted herein as Ru_1_Pt_1_ and Ru–H, respectively, to more precisely reflect the nominal concentrations associated with and synthetic protocol used in their generation) were produced using a previously reported hydrothermal synthesis method,^57,58^ so that we could compare these samples with the NWs synthesized by our in-house protocol. In this prior procedure, RuCl_3_ and H_2_PtCl_6_ were reacted with polyvinylpyrrolidone (PVP), sodium dodecyl sulfate (SDS), and NaBr in water under hydrothermal conditions. Whereas PVP behaved as a mild reducing agent and surfactant, the role of SDS was that of an additional surfactant, while NaBr functioned as a structure-directing agent. As such, the ultrathin NWs grew within as-formed soft templates, mediated by the presence of SDS and PVP. The as-generated Ru_1_Pt_1_ and Ru–H NWs yielded average diameters of 3.0 ± 0.3 and 3.4 ± 0.4 nm, respectively, as can be observed in Fig. S2C and D.† The *d*-spacing values, measured from the HRTEM images in Fig. 1C and D, were determined to be 0.21 and 0.23 nm, which are within experimental error for what would be expected for *hcp* Ru and *fcc* Ru_1_Pt_1_, respectively.^52,57^

XRD patterns of the Ru_1_Pt_1_ and Ru–H NWs shown in Fig. S3A† are indicative of the formation of the expected *fcc* and *hcp* structure for Ru_1_Pt_1_ and Ru–H, respectively. In addition, in accordance with Vegard's law, the shift to higher values of 2*θ* in the XRD pattern for Ru_1_Pt_1_, as compared with the Pt reference standard, is consistent with alloy production, namely the incorporation of Ru within the underlying *fcc* Pt lattice. Moreover, whereas equimolar amounts of the two metal precursors with a Ru : Pt ratio of about 1 : 1 were used to generate the Ru_1_Pt_1_ NWs, the actual chemical composition of our Ru_1_Pt_1_ sample was ascertained using quantitative EDS, which was consistent with a Ru : Pt ratio of 26 : 74. Elemental mapping results, shown in Fig. 2E and F, indicate that while there is some spatial overlap between the Ru and Pt signals, it is imperfect and that there is likely some degree of elemental segregation, in accordance with the idea of a heterogeneous alloy formation in this case. In fact, the corresponding EDS line-scan profile (Fig. S4B†) of Ru_1_Pt_1_ NWs, characterized by the lack of any spatial coincidence between Ru and Pt, corroborates the likelihood of the presence of a heterogeneous alloy, comprised of Pt-rich regions with low Ru content.

#### Pt and Pd-based NWs

A previously reported and reliable ‘soft-template’ procedure was utilized to generate not only the compositionally distinctive series of Pd_1_Pt_1_ and Pd_1_Pt_9_ NWs but also their mono-metallic Pd and Pt NW counterparts.^53^ This method relies on the formation of hexadecyltrimethylammonium bromide (CTAB) micelles, produced within a mixture of chloroform and water. The reduction of the metal precursors within the micellar templates is initiated by the addition of the strong reducing agent, NaBH_4_. In this synthesis, the chemical compositions of the NWs could be somewhat controlled by adjusting the molar ratio between the two metal precursors. For all ultrathin NWs produced, regardless of the synthetic procedure used, it was a challenge to directly correlate the incident precursor concentrations with the resulting elemental stoichiometry in these systems.

TEM images (Fig. S2E–H†) are consistent with the formation of ultrathin NWs with average diameters of 3.1 ± 0.4, 5.5 ± 1.1, 4.0 ± 1.0, and 3.0 ± 0.4 nm for Pt, Pd, Pd_1_Pt_9_, and Pd_1_Pt_1_ NWs, respectively. Moreover, the associated HRTEM images (Fig. 1E–H) highlight not only a *d*-spacing of 0.22 nm for the Pd NWs alone but also *d*-spacings of 0.23 nm that are consistent with the production of Pt, Pd_1_Pt_1_, and Pd_1_Pt_9_ NWs, respectively. XRD analysis of the PdPt samples confirms the expected *fcc* structure. In terms of isolated stoichiometries generated, a point which emphasizes the difficulty of precisely controlling ultrathin NW composition, the actual Pd : Pt ratios within the predicted Pd_1_Pt_1_ and Pd_1_Pt_9_ samples were measured to be 65 : 35 and 3 : 97 by quantitative EDS, respectively. The complementary elemental mapping data set indicates that there is an even and spatially uniform distribution of the elements, consistent with the formation of bimetallic alloys, as shown in Fig. 2H–L. Indeed, this finding is further confirmed by the EDS line scans for the PdPt NW data set (Fig. S4C and D†), especially Pd_1_Pt_1_, wherein, unlike what had been observed for the analogous Ru_1_Pt_1_ NWs, we noted a spatial overlap of the elements, implying a relatively homogeneous distribution of Pt with Pd.

#### Au and Ag-based NWs

As mentioned, Au and Au_1_Ag_1_ NWs were produced using a method,^54^ wherein the metal precursors are reduced by KBH_4_ and in parallel, Triton X-114 is used as a capping agent to direct the growth of ultrathin NWs. The average diameters of the as-prepared Au and Au_1_Ag_1_ NWs were measured to be 5.3 ± 1.2 and 5.5 ± 0.9 nm, respectively, as determined by TEM (Fig. S2I and J†). The *d*-spacings, measured by HRTEM (Fig. 1I and J), were determined to be 0.24 nm for both the Au and Au_1_Ag_1_ NWs, respectively, corresponding to the expected value for the (111) plane of not only Au but also Ag. The chemical composition and crystallinity of the two NWs were further probed by XRD (Fig. S3C†), and corroborate the finding that *fcc* Au and Au_1_Ag_1_ were produced. With respect to chemical composition, despite the use of a 1 : 1 molar precursor ratio of Au to Ag, quantitative EDS measurements of these Au_1_Ag_1_ NWs elucidated an actual Au : Ag ratio of 72 : 28. Nevertheless, complementary EDS mapping data in Fig. 2N and O also suggested that a homogeneous bimetallic alloy had in fact formed. This assertion is substantiated by the associated EDS line-scan profile (Fig. S4E†).

### EXAFS analysis

#### Description of models analyzed

There are various different structural configurations that can be found in a bimetallic system. One such architecture involves the formation of a segregated structure, wherein there are particles consisting only of A atoms and particles containing solely B atoms, in which there is no alloying between the two metals. The opposite of a segregated model is one in which there is interaction between two different metals to form an alloy.

There are a number of interesting structural possibilities associated with alloy generation. These include (i) the formation of a homogeneous alloy structure, (ii) a homophilic structure, and (iii) a core–shell configuration. Within a (i) homogeneous alloy structure, a given (A- or B-) atom type maintains the same number of neighbors, on average, thereby indicating a homogeneous, uniform distribution of atoms of different types within the alloy. (ii) A homophilic structure gives rise to clustering of ‘like’ atoms, wherein there are A- or B-rich regions, but there is still mixing of the two different atom types. In a (iii) core–shell structure, one atom type segregates at the surface, whereas the other is localized within the center core. Surface atoms possess fewer nearest neighbors, thereby reducing their average coordination number, as measured by EXAFS.^49,65^ The coordination numbers obtained from the EXAFS analysis for a given bimetallic system can be used, along with the known concentrations of the constituent metals, in order to determine which of the aforementioned structural configurations actually formed experimentally.

One methodology for enabling the determination of the local structure within a bimetallic structure involves the use of Cowley's short range order parameter, *α*,^48,66–68^ which can be used to assess the homogeneity of a given bimetallic system. It is expressed, as follows (eqn (1)):1
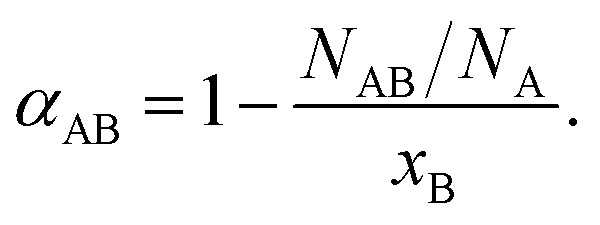
In the above equation, *N*_AB_ represents the first nearest neighboring A–B coordination number; *N*_A_ is the total coordination number for A-metal neighbors; and *x*_B_ denotes the concentration of the atomic species B. The coordination numbers can be used to determine the relationship of *x*_B_/*x*_A_ of a bimetallic system by taking the ratio of *N*_AB_ and *N*_BA_:^36^2
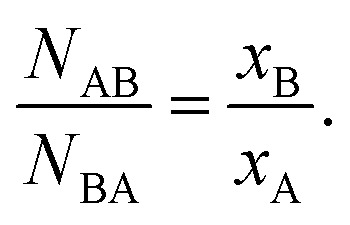


It should be noted that the same ratio, as shown in the right side of eqn (1), was also used in the analogous procedure reported by Hwang and co-workers. Not surprisingly, the conclusions that are presented here on the basis of the *α* parameter are identical to what one would have obtained using the conceptually identical methodology described by Huang *et al.*^69^

Using 2D alloys as an illustration,^48^ if *α*_AB_ = −1, then there exists perfect long-range order, wherein atoms of type A maintain equivalent surroundings by the opposite atoms only. If −1 < *α*_AB_ ≤ 0, then the atoms of type A are preferentially surrounded by atoms of type B and *vice versa*, suggestive of a negative tendency towards the clustering of ‘like’ atoms. If *α*_AB_ = 0, then a random alloy is formed, meaning that for either atom type, the probabilities for the neighboring atoms to consist of either A- or B-type atoms are partitioned, according to their relative concentrations in the alloy. If 0 < *α*_AB_ ≤ 1, then there is a positive tendency for the clustering of ‘like’ atoms. In the specific case of *α*_AB_ = 1, a completely segregated structure is predicted, implying that there is no mixing between the two atom types (*N*_AB_ = 0). It should be noted that in all of the above cases, values for *α*_AB_ and *α*_BA_ should be precisely equal to each other under the condition that *N*_A_ = *N*_B_, a result that follows from eqn (1) and (2). Therefore, prior to determining the *α* parameter, one should first investigate the relationship between the total coordination numbers *N*_A_ and *N*_B_ of the two different elements.

If *N*_A_ and *N*_B_ are equal, then no element has a preference to surface (or core) segregation. However, if *N*_A_ is greater than *N*_B_, then the A atoms are more likely to be located in the core, whereas the B atoms are likely to segregate to the surface. If one were to calculate the *α*_AB_ for this case, then the values for *α*_AB_ and *α*_BA_ would not be identical to each other, indicative of a scenario in which there is not good mixing between the two atom types. In other words, when the distribution of different atomic types is not homogeneous, wherein the degree of homogeneity can be verified by comparing the *N*_A_ and *N*_B_ values, as discussed above, the *α* parameter cannot be dependably used to quantify the short range order (degree of mixing) of the elements.

#### General comments

**Fig. 3:**
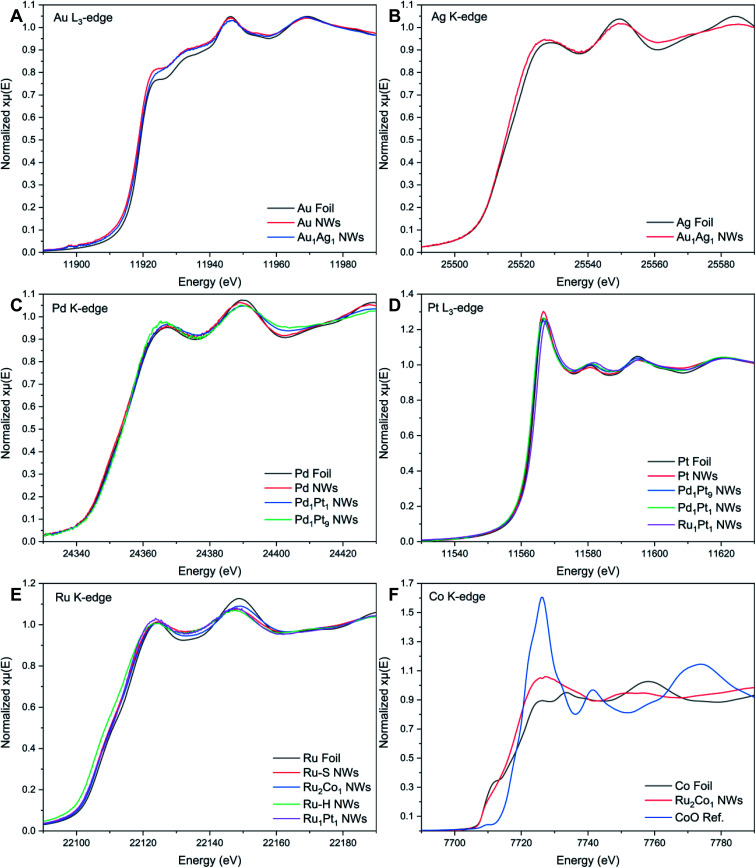
XANES spectra associated with the (A) Au L_3_-edge, (B) Ag K-edge, (C) Pd K-edge, (D) Pt L_3_-edge, (E) Ru K-edge, and (F) Co K-edge, respectively.

XAS measurements were collected of the various NWs in order to investigate the local atomic and electronic structures. Fig. 3 highlights the XANES spectra of the various NW systems, along with the reference foils, associated with the different edges.

The Au L_3_-edge absorption edge for Au_1_Ag_1_ is shifted to higher energies as compared with that of the monometallic Au NWs, thereby indicating that Au likely donates electrons to Ag (Fig. 3A). The similarity of features at the Au and Ag edges within the alloy NWs by comparison with their respective foils (Fig. 3A and B) signifies that both constituent components of the nanowire alloy likely exist in the metallic state. In addition, the absorption features for the Pd, Pt, Pd_1_Pt_1_, and Pd_1_Pt_9_ NWs at both the Pd K- and Pt L_3_-edges (Fig. 3C and D) are consistent with the metallic characteristics of all of the elements.

**Fig. 4:**
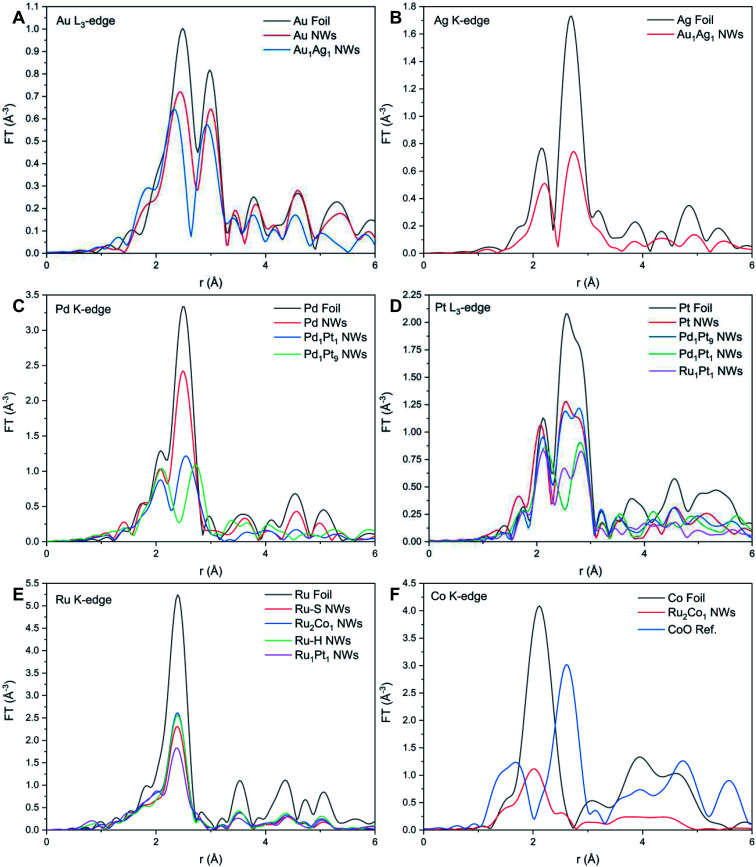
Fourier transforms of *k*^2^*χ*(*k*) spectra associated with the (A) Au L_3_-edge, (B) Ag K-edge, (C) Pd K-edge, (D) Pt L_3_-edge, (E) Ru K-edge, and (F) Co K-edge, respectively.

**Fig. 5:**
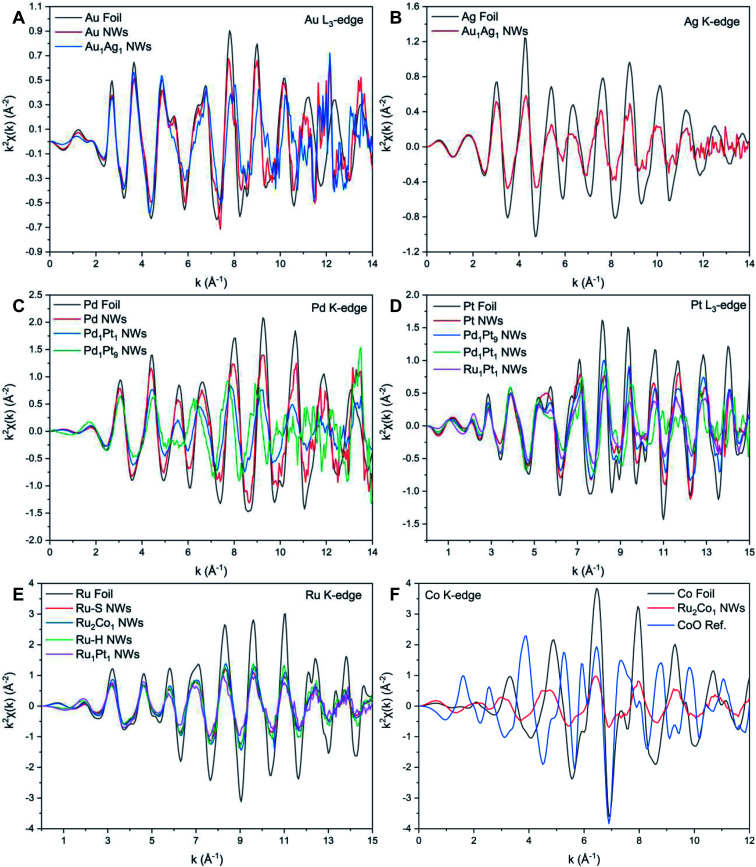
*k* ^2^-Weighted EXAFS data for the (A) Au L_3_-edge, (B) Ag K-edge, (C) Pd K-edge, (D) Pt L_3_-edge, (E) Ru K-edge, and (F) Co K-edge, respectively.

By comparison, XANES spectra collected at the Ru K- and Pt L_3_-edges for Ru_1_Pt_1_, Ru–H, and Ru–S NWs also point to the metallic state of all of these materials. For Ru_2_Co_1_, the spectrum at the Ru K-edge (Fig. 3E) closely resembles that of the Ru–S NWs, again suggesting that Ru exists in its metallic state. However, the XANES spectrum at the Co K-edge (Fig. 3F) for the Ru_2_Co_1_ NWs differs from the corresponding spectrum of either the Co foil or even a CoO reference, denoting unusual behavior with respect to the other elements we have analyzed. In fact, the XANES spectrum for our Ru_2_Co_1_ NWs is much more similar to that which has been previously reported for Co-substituted Ru nanosheets possessing a similar composition.^27^ It is unlikely that the NWs exist within an oxidized state, since the absorption onset is close to that observed for the Co foil. Indeed, the broadening of features and the concomitant decrease in the magnitude of such features at higher energies, thereby resulting in the spectral differences observed, can likely be collectively attributed to a high degree of disorder in the local atomic structure, especially as compared with the corresponding metallic foil.^70,71^ In order to further investigate the local atomic structure of our various systems, we have analyzed their EXAFS spectra (Fig. 4 and 5).

#### Au and Au–Ag

Table S1 and Fig. S6† present the fitting results for the Au and Au_1_Ag_1_ NWs. The EXAFS analysis for both the Au L_3_- and Ag KK-edges (Fig. 4A and B)-edges confirms that there is no apparent oxidation of either of these elements within the NWs. For the monometallic Au NWs, the smaller *N*_Au–Au_ value, as compared with that for the Au foil, provides evidence of the NW nanoscale size. The total *N*_Au_ for the Au_1_Ag_1_ NWs was constrained to be equal to the *N*_Au–Au_ for the Au NWs, so as to prevent the fit from yielding a *N*_Au_ larger than 12, which is not physically possible. Making this assumption about the equivalence of the total coordination number of Au between the Au and Au_1_Ag_1_ NWs is reasonable, because the sizes of these two NWs are similar in magnitude, as demonstrated by TEM analysis.

While it is well known that Au and Ag satisfy the Hume-Rothery rules and thus are expected to form completely miscible alloys in the solid phase at the bulk scale,^72^ this is not what we have observed herein with our bimetallic system. Specifically, by comparing the *N*_Au_ and *N*_Ag_ values for Au_1_Ag_1_, wherein *N*_Au_ (10.3) is notably larger than that of *N*_Ag_ (8.8), while taking into account of the experimental error bars, it is proposed that there is segregation of the two elements within the NWs. In this picture, Au tends to be confined to the center, whereas Ag segregates at the external surface. Indeed, since these results indicate that there is segregation of Au and Ag within the NWs, the calculation of the short-range order *α* parameters cannot be reliably used to characterize the short-range order of the samples. In fact, calculating *α*_Au–Ag_ and *α*_Ag–Au_ yields dissimilar values of 0.26 and 0.14, respectively. This finding is fully consistent with the conclusion of elemental segregation, discussed earlier, which had been made on the basis of a comparison of the corresponding *N*_Au_ and *N*_Ag_ values. However, it should be noted that the EXAFS analysis does not imply either full, complete elemental segregation or the creation of a perfectly generated core–shell configuration. At most, we can postulate the formation of a motif, comprised of an Ag-enriched ‘shell’ coupled with an Au-enriched ‘core’. A similar observation regarding the possibility of some degree of elemental mixing (*i.e.* partial alloying), occurring within the context of a core–shell bimetallic NP system consisting of Au and Ag, has been previously reported.^73^ By contrast, the EDS mapping and associated EDS line-scan results, shown in Fig. 2M–O and S4E,† collectively suggest the presence of a reasonably even distribution of the constituent elements, with no clear evidence for core–shell formation. As such, this apparent discrepancy between EXAFS and EDS findings in accounting for elemental distribution highlights issues with reconciling the localized composition within ultrathin NWs with data ‘averaged’ across the entire sample.

It has been reported that segregation within nanostructures can be caused by factors, related to surface energy, size, strain effects, and charge transfer between atoms.^74,75^ There are likely no strain effects, which lead to the apparent segregation within Au_1_Ag_1_, because the lattice constants for Ag and Au are very similar. Moreover, charge transfer between Au and Ag would favor mixing between the two elements, a scenario which would have reduced the tendency of Ag to selectively segregate at the surface. In effect, the XANES spectrum for the Au absorption edge (Fig. 3A) suggests that there is in fact charge transfer occurring between Au and Ag, which therefore ought to promote mixing.

As such, we hypothesize that there must be another factor that accounts for the observed segregation. One such parameter is the greater surface energy of Au *versus* that of Ag, a variable which is conducive to the surface segregation of Ag.^76^ In addition, it has been reported that the reaction temperature can have a significant influence upon the segregation of Au and Ag, wherein the surface enrichment of Ag tends to be favored at lower temperatures.^77,78^ As such, the evident formation of Au–Ag core–shell motifs herein is likely promoted by our synthetic protocol, because our ultrathin NWs were generated at relatively reduced temperatures of ∼0 °C.

#### Pt and Pt–Pd

The EXAFS fitting analysis results for the series of as-prepared PdPt NW samples are summarized and displayed in Table S2 and Fig. S7.† The lack of either Pt–O or Pd–O peaks within the *r*-space (Fig. 4C and D) of all of NW samples implies that there are no oxidative species present in the XANES spectra. However, for the monometallic Pt samples, we noted contributions ascribable to Pt–Cl species, which are likely due to the presence of some unreacted H_2_PtCl_6_ precursor. For both PdPt alloy NW samples, the values of *N*_Pd_ and *N*_Pt_ are within the standard deviation of each other, which suggests that there is no significant segregation, unlike what had been observed for AuAg. In this case, the XANES data, which validate the notion of alloy formation, are consistent with the elemental mapping and corresponding EDS line-scan data, presented in Fig. 2H–L and S4C and D,† respectively. Indeed, with Pd_1_Pt_1_, the likely presence of alloying is substantiated by the nearly identical bond lengths associated with Pt–Pt, Pd–Pt, and Pd–Pd pairs. Therefore, the short-range order parameter can be calculated for these NW samples in order to determine the randomness factor in these alloys.

The *α* values for both PdPt samples are presented in Table 1. For both Pd_1_Pt_1_ and Pd_1_Pt_9_, the values for *α*_Pt–Pd_ and *α*_Pd–Pt_ are approximately equal to each other, as expected, but not identical. In fact, *α* = 0.07 for Pd_1_Pt_9_; because this number is close to 0, it would be associated with the generation of a random alloy. With Pd_1_Pt_1_, the slightly larger values for *α*_Pt–Pd_ (0.25) and *α*_Pd–Pt_ (0.27) are indicative of a slight tendency towards clustering. However, conversely, these positive values may be caused by compositional polydispersity, a consequence of which is that a random, homogeneous alloy may be mistaken for a heterogeneous system.^48^ These findings suggest that it is not necessarily possible to conclusively differentiate between either intra-particle or inter-particle segregation using EXAFS alone.

Bimetallic compositions as obtained by EDS and EXAFS, comparison of the total coordination numbers, and short-range order parameters, *α*. Uncertainties in the last digits are displayed in parentheses

**Table:** 

Sample	AuAg NWs						
	*x*_Ag_/*x*_Au_		*N*_Au_ *vs. N*_Ag_	*α*_Au–Ag_ a		*α*_Ag–Au_ a	
	EDS^b^	EXAFS^c^		EDS^d^	EXAFS^e^	EDS^d^	EXAFS^e^
Au_1_Ag_1_	0.4	0.6(2)	10.3(1.3) > 8.8(1.0)	n/a	n/a	n/a	n/a

a Calculated using eqn (1). If −1 ≤ *α* ≤ 0 then there is a tendency for alloying, whereas if 0 < *α* ≤ 1 then there is a tendency for clustering of ‘like’ atoms. It should be noted that if a computed value were to fall outside of this range, then it is likely that the underlying data used do not accurately reflect the nature of the entirety of the sample.

b Determined on the basis of quantitative EDS measurements.

c Calculated using eqn (2).

d Calculated with eqn (1) using the concentrations which had been determined with quantitative EDS measurements.

e Calculated using eqn (1) with the concentrations derived from both EXAFS analysis and eqn (2).

**Table:** 

Sample	PdPt NWs						
	*x*_Pd_/*x*_Pt_		*N*_Pd_ *vs. N*_Pt_	*α*_Pt–Pd_ a		*α*_Pd–Pt_ a	
	EDS^b^	EXAFS^c^		EDS^d^	EXAFS^e^	EDS^d^	EXAFS^e^
Pd_1_Pt_1_	1.8	0.7(1)	9.4(7) ≈ 9.2(7)	0.51	0.25	−0.19	0.27
Pd_1_Pt_9_	0.03	0.10(4)	9.0(2.2) ≈ 9.0(4)	−2.05	0.07	0.13	0.07

**Table:** 

Sample	Ru-based NWs						
	*x*_M_/*x*_Ru_		*N*_M_ *vs. N*_Ru_	*α*_Ru–M_ a		*α*_M–Ru_ a	
	EDS^b^	EXAFS^c^		EDS^d^	EXAFS^e^	EDS^d^	EXAFS^ee^
Ru_2_Co_1_	0.2	0.2(2)	9.5(2.0) ≈ 8.9(8)	0.73	0.75	0.77	0.77
Ru_1_Pt_1_	2.8	1.2(8)	8.7(6) ≈ 7.9(7)	0.81	0.75	0.61	0.77

Nonetheless, the case for proposing that our PdPt NWs exhibited a broad compositional distribution is supported by the difference in chemical compositions, as measured by quantitative EDS and EXAFS, with the data summarized in Table 1. For example, Pd_1_Pt_1_ NWs were measured to have a *x*_Pd_/*x*_Pt_ value of 1.8 by quantitative EDS analysis, which is much larger than the corresponding value of 0.7, as determined by EXAFS. We would expect the measurement determined by EXAFS to be a better representative of the ensemble average of the entire sample, whereas EDS is a superior indicator of local behavior associated with a smaller region within the sample. To further reinforce this idea, we compared the *α* parameters, calculated by using the chemical compositions determined by EXAFS and EDS, respectively, as shown in Table 1.

As previously mentioned, the values for *α*_Pt–Pd_ and *α*_Pd–Pt_, as determined by using the EXAFS composition, are approximately equal to each other for both PdPt NW samples. However, this is not the case for *α*_Pt–Pd_ and *α*_Pd–Pt_ calculated using the analogous EDS data, thereby hinting at the presence of significant segregation. Moreover, the value for *α*_Pt–Pd_ associated with Pd_1_Pt_9_, as determined by EDS data, was found to be approximately −2, which is not a physically meaningful quantity. Hence, a logical inference would be that the EDS results, collected on a small, localized region, do not accurately convey the compositional picture and associated nuances of the entire area of the overall sample.

Specifically, we should emphasize that the values calculated for *x*_Pd_/*x*_Pt_, as derived from EXAFS, namely 0.7 for Pd_1_Pt_1_ and 0.10 for Pd_1_Pt_9_, are much closer to their nominal, expected compositions. By contrast, the analogous compositional figures, calculated using the chemical composition determined by EDS, are 1.8 for Pd_1_Pt_1_ and 0.03 for Pd_1_Pt_9_. This apparent discrepancy between the data emanating from these two compositional analysis techniques suggests that there is likely a broader, more polydisperse distribution of chemical compositions within the NWs themselves, which can thereby impact the ostensible calculated *α* values. These results highlight the apparent shortcomings of exclusively relying upon EDS data, collected from a small region of the sample, as an accurate means for providing definitive conclusions about the overall sample composition. Nonetheless, in this case, the notion of visualizing our as-prepared PdPt NWs as homogeneous alloys is corroborated by the combination of data from our EXAFS analysis coupled with EDS mapping measurements.

#### Ru, Ru–Pt, and Ru–Co

Table S3 and Fig. S8† present the EXAFS fitting results for the Ru–H and Ru_1_Pt_1_ NWs generated by the hydrothermal method. Again, the absence of any oxide peaks within the FT-EXAFS signal (Fig. 4D and E) suggests that the metals occur within a metallic state. By noting the similarity of the *N*_Ru_ and *N*_Pt_ values, we can reasonably conclude that there is no surface segregation of either element. Hence, we can use the short-range order parameter to determine if the NWs are either homogeneous or heterogeneous in nature, as shown in Table 1.

Both the *α*_Ru–Pt_ (0.75) and *α*_Pt–Ru_ (0.77) values, obtained from the EXAFS results, are largely positive, which indicates that there is a tendency for clustering of ‘like’ atoms; this finding corresponds well with the elemental mapping data, shown in Fig. 2. Moreover, the EDS-derived values of *α*_Ru–Pt_ (0.81) and *α*_Pt–Ru_ (0.61), while still largely positive, also suggest an inclination towards some degree of aggregation of ‘like’ atoms. Nevertheless, as with the PdPt NWs, it should be noted that there may be a broad compositional distribution, as indicated by the difference in the apparent chemical compositions extrapolated from EDS and EXAFS data, respectively. By analogy to what was observed with the PdPt samples, the compositions determined by EXAFS for *x*_Pt_/*x*_Ru_ of 1.2 is closer to the nominal composition than what was determined by EDS (*x*_Pt_/*x*_Ru_ of 2.8). Nonetheless, as mentioned previously, the *α* values herein are close to 1. Coupled with the elemental mapping and associated EDS line-scan data, overall, some heterogeneity is likely present; one manifestation would be the spatial aggregation of ‘like’ atoms within Ru_1_Pt_1_.

Table S4 and Fig. S8† reveal the EXAFS fitting results for both Ru–S and Ru_2_Co_1_ NWs. In parallel with the results associated with the other NW systems, the FT-EXAFS signal (Fig. 4E and F) does not show the presence of any oxidative species, thereby implying that the individual metals exist within their metallic state. The decrease in *R*_Ru–Ru_ and concomitant increase in *R*_Co–Ru_ as compared with Ru NWs and reference foils collectively signify that there is some degree of alloying between the two elements. The comparable values of *N*_Ru_ and *N*_Co_ within Ru_2_Co_1_ suggests that there is no surface segregation. Hence, we used the short-range order parameter to investigate the distribution of elements, as shown in Table 1.

By analogy to what had been observed for Ru_1_Pt_1_, the *α* values are largely positive, which would imply that there is some degree of agglomeration of ‘like’ atoms. However, unlike what had been observed for PdPt and Ru_1_Pt_1_, the chemical compositions derived from the quantitative EDS and EXAFS measurements are in agreement with one another. Both data sets advocate for a high degree of heterogeneity within these Ru_2_Co_1_ NWs. Interestingly, the sum of elemental mapping and EDS line-scan results, shown in Fig. 3 and S4A,† respectively, reveals the opposite scenario, namely a relatively even and uniform distribution of Ru and Co, which is clearly different from what had been found for Ru_1_Pt_1_.

As we have seen, with Ru_1_Pt_1_, the presence of heterogeneity was consistent with the findings of both EDS and EXAFS analyses. Hence, the key difference between Ru_1_Pt_1_ and Ru_2_Co_1_ can likely be ascribed to their divergent chemical compositions. Specifically, the area occupied by Co clusters within Ru_2_Co_1_ is likely to be smaller in size by comparison with analogous Pt clusters within Ru_1_Pt_1_, because of the lower concentration of Co in the former. Overall, these results would signify that whereas EDS mapping can be used to detect heterogeneity within bimetallic alloys possessing nearly equal concentrations of the two constituent elements, as observed with Ru_1_Pt_1_, it is less effective at detecting heterogeneity within comparable alloys, characterized by a relatively lower concentration of one of the component metals. Hence, as with the PdPt NW series, we show that for the Ru-based systems, the intrinsic advantage of EDS mapping in providing detailed chemical compositional information about a spatially localized area can actually be detrimental to achieving accurate and meaningful insights into the compositional traits of the entire sample, for which it is therefore necessary to acquire complementary EXAFS data.

## 3. Context and conclusions

In this work, we have reported on a unique, facile, and readily generalizable synthesis of ultrathin Ru_2_Co_1_ NWs, possessing average diameters of 2.3 ± 0.5 nm, obtained through the mediation of the simultaneous use of both OAm and OAc as surfactants with which to guide the growth of the NWs. Furthermore, we have investigated the local atomic structure of these novel Ru_2_Co_1_ NWs, in conjunction with analogous ultrathin Ru_1_Pt_1_, Au_1_Ag_1_, Pd_1_Pt_1_, and Pd_1_Pt_9_ NWs which were generated as ‘controls’. Specifically, we have calculated structure-dependent *α* parameters for each sample as a means of characterizing the distribution of elements throughout the alloyed nanowires. In so doing, we have determined that both Ru-based NWs maintain a strong tendency for clustering of ‘like’ atoms, as indicated by their largely positive *α* parameters. By contrast, the *α* parameter calculated for Pd_1_Pt_9_ is close to 0, suggesting that a random alloy likely formed, whereas the corresponding value calculated for Pd_1_Pt_1_ is slightly positive, implying a small degree of aggregation of ‘like’ atoms. With Au_1_Ag_1_ NWs, the EXAFS results are consistent with the creation of a core–shell structure, consisting of an Au-rich core and an Ag-rich shell; indeed, *N*_Au_ is significantly larger than *N*_Ag_, and equivalently, *α*_Au–Ag_ and *α*_Ag–Au_ are sufficiently dissimilar, such that either of these data corroborate the presence of segregation.

A key motivation for our study is that we have been fundamentally interested in the question of the interplay between quantitative EDS, EDS mapping (with associated line scans), and EXAFS in yielding valuable and reliable structural insights into the formation of ultrathin NWs. We have found that basing conclusions using either TEM-based EDS or EXAFS in and of itself is insufficient, because each compositionally distinctive NW system is unique.

For example, the spatially localized EDS mapping of both PdPt samples, regardless of stoichiometric composition, was consistent with the formation of a homogeneous PdPt alloy, a picture which was further backed up by EXAFS analysis. By contrast, the results for our as-prepared Au_1_Ag_1_, Ru_1_Pt_1_, and Ru_2_Co_1_ NWs suggest inherent limitations, due to apparent sensitivity issues, associated with relying on EDS mapping alone for establishing the presence of surface segregation or heterogeneity within bimetallic systems. In the case of Au_1_Ag_1_, localized, spatially confined elemental mapping was not sufficient in determining if there was any surface segregation, the evidence of which was actually provided by EXAFS analysis. Elemental mapping can indicate heterogeneity and yield results which agree with those from EXAFS analysis in bimetallic samples possessing equal concentrations of both elements, as observed in the case of Ru_1_Pt_1_ NWs. However, the use of EDS mapping alone is inadequate in assessing the heterogeneity within a bimetallic alloy incorporating a low concentration of either one of the elements involved, as noted in the example of Ru_2_Co_1_ NWs, for which EXAFS analysis coupled with quantitative EDS implied the presence of a significant degree of clustering of ‘like’ atoms.

Moreover, we have observed that relative to TEM-based EDS, EXAFS is more accurate and effective in determining the chemical composition of a whole sample. These findings were especially evident when analyzing not only Ru_1_Pt_1_ but also our series of compositionally distinctive PdPt NWs. In these examples, EXAFS analysis yielded measured values for the chemical composition, that were closer to the nominal composition expected for these material systems based on the precursor quantities used in their syntheses. As a salient demonstration, with Pd_1_Pt_9_, calculating *α*_Pt–Pd_ using the composition measured by EDS generated a largely negative value of approximately −2, which means nothing because it has no physical basis in reality. Similarly, for both Pd_1_Pt_1_ and Ru_1_Pt_1_, the EDS-derived *α*_AB_ and *α*_BA_ parameters were significantly different from one another; by contrast, the analogous values determined from EXAFS results were more consistent with each other, did not imply any significant elemental segregation, and likely reflected the composition of the sample taken in its entirety. This apparent discrepancy between EDS and EXAFS is likely due to the fact that EDS samples a relatively small region, whereas EXAFS with its higher flux of irradiation enables a more accurate, ‘averaged’ assessment over a larger sample area.

Hence, overall, it is clear that only a systematic comparison and favorable convergence of EDS and EXAFS results can provide for a true and valid representation of the chemical and structural nuances associated with various bimetallic ultrathin NW systems. Specifically, to overcome limitations in interpretation, there is a need to reconcile the localized EDS information with the ensemble averaged picture provided by EXAFS of the distribution of atoms within a bimetallic system. Moreover, while the quantitative EXAFS models themselves that we studied herein tend to be mutually consistent in their overall conclusions, they still need to be improved upon and optimized in order to properly acquire a thorough and accurate understanding of local atomic structure, especially for predicting and correlating electrocatalytic performance within relatively complex systems, such as ultrathin bimetallic NW alloys.

## 4. Experimental section

### Materials

All chemicals were used without further purification. These included ethanol (denatured, BeanTown Chemical), gold chloride (AuCl_3_, Au 64.4% min, Alfa Aesar), silver nitrate (AgNO_3_, 99.9%, J.T. Baker Chemical Company), Triton X-114 (reagent grade, VWR), hexadecyltrimethylammonium bromide (CTAB, ≥98%, Sigma Aldrich), chloroform (99.8+%, Alfa Aesar), sodium tetrachloropalladate (Na_2_PdCl_4_, 99.9%, Alfa Aesar), sodium borohydride (NaBH_4_, 99.99%, Sigma Aldrich), potassium borohydride (KBH_4_, 98%, Alfa Aesar), polyvinylpyrrolidone (PVP, MW: 40k, Alfa Aesar), sodium dodecyl sulfate (SDS, Biotechnology Grade, VWR), sodium bromide (NaBr, 99.4%, J.T. Baker Chemical Co.), dihydrogen hexachloroplatinate(iv) hydrate (H_2_PtCl_6_, 99.9%, Alfa Aesar), ruthenium chloride (RuCl_3_ 99.9%, BeanTown Chemical), cobalt acetate tetrahydrate (Co(OOCCH_3_)_2_, 98%, Alfa Aesar), oleic acid (OAc, 90%, Alfa Aesar), and oleylamine (OAm, 70%, Sigma Aldrich).

### Synthesis protocols

#### Synthesis of PdPt, Pd, and Pt NWs

Monometallic Pd and Pt NWs, along with the associated bimetallic NWs, were synthesized using a previously reported, dependable soft-template method.^52,53^ With respect to the synthesis of Pt NWs, an aqueous solution of H_2_PtCl_6_ (5 mL, 20 mM) was combined together with 5 mL of a 40 mM CTAB surfactant solution in chloroform. Next, 40 mL of water was added, and the mixture was stirred for 30 min. Separately, 0.2 g of NaBH_4_ was dissolved in 5 mL of water, which was then inserted to the reaction mixture, with stirring; this mixture was allowed to react for 20 min. The NWs were collected by centrifugation, and subsequently washed with water and ethanol. The analogous Pd NW synthesis was identical to that of the Pt NWs, except for the substitution of a Na_2_PdCl_4_ solution *in lieu* of H_2_PtCl_6_.

The bimetallic NW series, prepared with nominal compositions of Pd_1_Pt_1_ and Pd_1_Pt_9_, were also synthesized using the identical methodology, but with the use of rationally chosen precursor molar ratios so as to generate the desired products with the projected stoichiometries. Specifically, for the synthesis of Pd_1_Pt_1_, aqueous solutions of H_2_PtCl_6_ (2.5 mL, 20 mM) and Na_2_PdCl_4_ (2.5 mL, 20 mM) were used. Similarly, in the corresponding synthesis of Pd_1_Pt_9_, aqueous solutions of H_2_PtCl_6_ (4.5 mL, 20 mM) and Na_2_PdCl_4_ (0.5 mL, 20 mM) were used.

#### Synthesis of Au and AuAg NWs

Au and Au_1_Ag_1_ NWs were generated using a different but nonetheless reliable protocol.^54^ In particular, in the synthesis of Au_1_Ag_1_ NWs, generated with an expected nominal concentration of 1 : 1, a 47 mL ice cold aqueous solution, containing 0.05 mmol AuCl_3_, 0.05 mmol AgNO_3_, and 25 mg Triton X-114 surfactant, was prepared within a 100 mL round bottom flask. The solution was stirred vigorously to which 3 mL of an ice-cold aqueous 0.1 M KBH_4_ reducing solution was rapidly injected. After 10 s, another 25 mg of Triton X-114 was added in. The solution was allowed to stir for 1 min, while kept at 0 °C. The NWs were collected by centrifugation and were washed three times with ethanol. The same procedure was utilized for the synthesis of monometallic Au NWs, wherein a total amount of 0.1 mmol AuCl_3_ was used as the sole metal-based precursor.

#### Synthesis of RuCo and Ru NWs by a surfactant-mediated method

We have developed a novel, ‘in-house’ procedure to produce RuCo and Ru NWs, which builds upon prior studies associated with the oleylamine-mediated synthesis of both pure Ru NPs^55^ and PtM (M = Co, Ni, Zn, Cu, Fe) NPs.^56^ Specifically, this synthesis was conducted under an inert atmosphere using typical, air-sensitive Schlenk line techniques.

First, a total of 0.25 mmol of metal precursors was added to 7.5 mL of OAm and 7.5 mL of OAc. RuCl_3_ and Co(OOCCH_3_)_2_ were used as the metal precursors with a Ru : Co molar ratio of 2 : 1; as such, this alloy is referred to herein as Ru_2_Co_1_ so as to reflect the predicted nominal composition. The solution was kept under argon gas, heated to 350 °C, and allowed to react for 1 h. The reaction was subsequently allowed to cool to room temperature and washed with mixtures of hexane, methanol, and ethanol, for several times. This identical procedure is utilized for the synthesis of Ru NWs by using 0.25 mmol of RuCl_3_ as the only metal precursor. Herein, the Ru NWs generated by this solution-based protocol are denoted as Ru–S NWs.

#### Synthesis of RuPt and Ru NWs by the hydrothermal method

RuPt NWs were fabricated by a previously reported hydrothermal method.^57,58^ Typically, 160 mg of PVP, 544 mg of SDS, 206 mg of NaBr, and a total of 0.2 mmol of H_2_PtCl_6_ and RuCl_3_ were dissolved in 15 mL of water and stirred for 30 min. The amounts of metal precursors initially used were determined by the desired chemical composition of the projected product. Herein, we used equimolar amounts of the two metal precursors to obtain NWs with a Ru : Pt ratio of about 1 : 1; hence, these NWs are described as Ru_1_Pt_1_ to indicate their anticipated chemical make-up. The solution was then transferred to a 20 mL Teflon lined autoclave and heated at 210 °C for 24 h. NWs were collected by centrifugation and washed with water and ethanol for several times. This same protocol was also used to generate monometallic Ru NWs by using the Ru precursor alone during the reaction process. Therefore, the Ru NWs synthesized using this hydrothermal procedure are referred to as Ru–H NWs.

### Structural characterization methods

#### X-ray diffraction (XRD)

Diffraction data were obtained using a Rigaku diffractometer, operating in the Bragg–Brentano configuration with Cu Kα_1_ irradiation (*λ* = 1.54 Å). All diffraction patterns were collected within the range from 10 to 90 degrees (2theta), using a scanning rate of 10 degrees per minute. In particular, powder samples were dispersed in ethanol and drop-cast onto a zero-background holder (MTI Corporation, zero diffraction plate for XRD, B-doped, p-type Si, 23.6 mm in diameter and 2 mm in thickness), followed by drying in air.

#### Transmission electron microscopy (TEM)

The morphology and size of all as-prepared ultrathin nanowire systems were probed using a JEOL 1400 TEM instrument. Low resolution TEM images were acquired using an accelerating voltage of 120 kV. High resolution transmission electron microscopy (HRTEM) images were taken with a JEOL 2100F instrument, which was operated with an accelerating voltage of 200 kV. Samples were prepared by drop casting onto 3 mm lacey carbon-coated copper grids, prior to analysis.

#### Scanning transmission electron microscopy (STEM); quantitative energy dispersive X-ray spectroscopy (EDS), and EDS mapping

High angle annular dark field scanning transmission electron microscopy (HAADF-STEM), along with associated EDS spectra and mapping data coupled with corroborating line scans, were acquired using a FEI Talos F200X instrument. Images were obtained using an acceleration voltage of 200 kV. Samples were prepared by initially dispersing in ethanol, followed by drop casting onto 3 mm lacey carbon-coated Cu grids.

#### XAS measurements

All XAS experiments were performed at the QAS (7-BM) beamline at the National Synchrotron Light Source II (NSLS-II) located in Brookhaven National Laboratory (BNL). All data were collected in the transmission mode. A double-crystal Si(111) monochromator was used to collect measurements at the Au L_3_-edge (11 919 eV), Ag K-edge (25 514 eV), Pt L_3_-edge (11 564 eV), Pd K-edge (24 350 eV), Ru K-edge (22 117 eV), and Co K-edge (7709 eV). Reference spectra for the corresponding metal foils were taken during each measurement to be used for energy calibration and data alignment. Data were processed and analyzed using the Athena and Artemis software packages.^59^ The Athena software was used to assign the photoelectron energy origin, *E*_0_, and to perform edge-step normalization and background subtraction of the measured X-ray absorption coefficient data. The background-subtracted and edge step-normalized absorption coefficient data were then transformed to *k* space. The *k*^2^-weighted data were subjected to Fourier transform (FT) to *r*-space, and EXAFS fitting was performed in *r*-space using Artemis.

Fitting was first performed on the EXAFS data of elemental metal foils, wherein the coordination number (*N*) of the first shell was set to be equal to 12, which is the expected value for all of the metals used herein. The passive electron reduction factors (*S*_0_^2^) were varied in the fit. For the bimetallic systems, multiple-edge analysis was done to fit the signals, measured from each of the alloying constituent component's absorption edge, simultaneously. Data for the monometallic samples were simulated using FEFF calculations performed using *fcc* structures for Pd, Pt, Ag, Au, and the *hcp* structure for Ru and Co. In order to calculate FEFF theory for the heterometallic samples incorporating elements A and B, the atoms of the type B were put into a first nearest neighbor position within the coordinate list with respect to the atoms of the type A. The *S*_0_^2^ parameters for the NWs were fixed to be equal to those obtained for the bulk foils.

For the bimetallic NWs, the fittings were performed for both edges concurrently, and the following constraints were applied.^36^ The heterometallic bond lengths were set to be equal (*R*_A–B_ = *R*_B–A_) along with the mean squared bond length disorders (*σ*_A–B_^2^ = *σ*_B–A_^2^), whereas the homometallic bond lengths (*i.e.*, *R*_A–A_ and *R*_B–B_) and mean squared bond length disorders (*i.e.*, *σ*_A–A_^2^ and *σ*_B–B_^2^) were varied independently. The coordination numbers were also modified independently for all samples, except for the Au_1_Ag_1_ NWs. For this latter sample, due to the relatively strong correlation of fitting parameters contributing to the amplitude of the EXAFS oscillations, the total coordination number for Au (*N*_Au_ = *N*_Au–Au_ + *N*_Au–Ag_) was set to be equal to 10.3, which was the coordination number calculated for the Au NWs.

## Author contributions

Conceptualization: S. S. W. and A. I. F. Formal analysis and data curation: S. C. M., A. M. E., N. H., L. Z., A. I. F., and S. S. W. Investigation, methodology, and validation: S. C. M., A. M. E., N. H., and L. Z. Project administration and supervision: S. S. W and A. I. F. Writing – original draft: S. C. M. and S. S. W. Writing – review & editing: S. C. M., A. M. E., N. H., L. Z., A. I. F., and S. S. W.

## Conflicts of interest

There are no conflicts of interest to declare.

## Supplementary Material

SC-012-D1SC00627D-s001

## References

[cit1] Scofield M. E., Liu H., Wong S. S. (2015). Chem. Soc. Rev..

[cit2] Li L., Tan S., Salvatore K. L., Wong S. S. (2019). Chem.–Eur. J..

[cit3] Li L., Wong S. S. (2018). ACS Omega.

[cit4] Liu H., Koenigsmann C., Adzic R. R., Wong S. S. (2014). ACS Catal..

[cit5] Liu H., Adzic R. R., Wong S. S. (2015). ACS Appl. Mater. Interfaces.

[cit6] Koenigsmann C., Santulli A. C., Gong K., Vukmirovic M. B., Zhou W.-p., Sutter E., Wong S. S., Adzic R. R. (2011). J. Am. Chem. Soc..

[cit7] Koenigsmann C., Scofield M. E., Liu H., Wong S. S. (2012). J. Phys. Chem. Lett..

[cit8] Koenigsmann C., Semple D. B., Sutter E., Tobierre S. E., Wong S. S. (2013). ACS Appl. Mater. Interfaces.

[cit9] Koenigsmann C., Sutter E., Adzic R. R., Wong S. S. (2012). J. Phys. Chem. C.

[cit10] Koenigsmann C., Sutter E., Chiesa T. A., Adzic R. R., Wong S. S. (2012). Nano Lett..

[cit11] Koenigsmann C., Wong S. S. (2011). Energy Environ. Sci..

[cit12] Koenigsmann C., Wong S. S. (2013). ACS Catal..

[cit13] Koenigsmann C., Zhou W.-p., Adzic R. R., Sutter E., Wong S. S. (2010). Nano Lett..

[cit14] Huang L., Zhang X., Wang Q., Han Y., Fang Y., Dong S. (2018). J. Am. Chem. Soc..

[cit15] Hu Y., Zhu A., Zhang Q., Liu Q. (2016). Int. J. Hydrogen Energy.

[cit16] Lu S., Eid K., Ge D., Guo J., Wang L., Wang H., Gu H. (2017). Nanoscale.

[cit17] Fu G., Yan X., Cui Z., Sun D., Xu L., Tang Y., Goodenough J. B., Lee J.-M. (2016). Chem. Sci..

[cit18] Huang L., Han Y., Zhang X., Fang Y., Dong S. (2017). Nanoscale.

[cit19] Li H.-H., Fu Q.-Q., Xu L., Ma S.-Y., Zheng Y.-R., Liu X.-J., Yu S.-H. (2017). Energy Environ. Sci..

[cit20] Li L., Liu H., Qin C., Liang Z., Scida A., Yue S., Tong X., Adzic R. R., Wong S. S. (2018). ACS Appl. Nano Mater..

[cit21] Pei J., Mao J., Liang X., Zhuang Z., Chen C., Peng Q., Wang D., Li Y. (2018). ACS Sustainable Chem. Eng..

[cit22] Zhang Y., Gao F., Song T., Wang C., Chen C., Du Y. (2019). Nanoscale.

[cit23] Jin Y., Chen F., Wang J. (2020). ACS Sustainable Chem. Eng..

[cit24] Bertella F., Lopes C. W., Foucher A. C., Agostini G., Concepcion P., Stach E. A., Martinez A. (2020). ACS Catal..

[cit25] Pirola C., Scavini M., Galli F., Vitali S., Comazzi A., Manenti F., Ghigna P. (2014). Fuel.

[cit26] Li W., Zhao Y., Liu Y., Sun M., Waterhouse G. I. N., Huang B., Zhang K., Zhang T., Lu S. (2021). Angew. Chem., Int. Ed..

[cit27] Mao J., He C.-T., Pei J., Chen W., He D., He Y., Zhuang Z., Chen C., Peng Q., Wang D., Li Y. (2018). Nat. Commun..

[cit28] Niu X., Tang Q., He B., Yang P. (2016). Electrochim. Acta.

[cit29] Wei Z., Liu Y., Peng Z., Song H., Liu Z., Liu B., Li B., Yang B., Lu S. (2019). ACS Sustainable Chem. Eng..

[cit30] Zhang F., Zhu Y., Chen Y., Lu Y., Lin Q., Zhang L., Tao S., Zhang X., Wang H. (2020). J. Mater. Chem. A.

[cit31] Wang H., Yang Y., Di Salvo F. J., Abruna H. D. (2020). ACS Catal..

[cit32] Gao D., Li H., Cheng X. (2015). ECS Trans..

[cit33] Li G., Zheng K., Li W., He Y., Xu C. (2020). ACS Appl. Mater. Interfaces.

[cit34] Feng T., Yu G., Tao S., Zhu S., Ku R., Zhang R., Zeng Q., Yang M., Chen Y., Chen W., Chen W., Yang B. (2020). J. Mater. Chem. A.

[cit35] Dobosz I., Kutyla D., Kac M., Wloch G., Zabinski P. (2020). Mater. Sci. Eng., B.

[cit36] Frenkel A. I. (2012). Chem. Soc. Rev..

[cit37] Knecht M. R., Weir M. G., Frenkel A. I., Crooks R. M. (2008). Chem. Mater..

[cit38] Merrill N. A., McKee E. M., Merino K. C., Drummy L. F., Lee S., Reinhart B., Ren Y., Frenkel A. I., Naik R. R., Bedford N. M., Knecht M. R. (2015). ACS Nano.

[cit39] Alayoglu S., Zavalij P., Eichhorn B., Wang Q., Frenkel A. I., Chupas P. (2009). ACS Nano.

[cit40] Sun J., Karim A. M., Zhang H., Kovarik L., Li X. S., Hensley A. J., McEwen J.-S., Wang Y. (2013). J. Catal..

[cit41] Hwang B.-J., Sarma L. S., Chen J.-M., Chen C.-H., Shih S.-C., Wang G.-R., Liu D.-G., Lee J.-F., Tang M.-T. (2005). J. Am. Chem. Soc..

[cit42] Nguyen T. S., McKeever P., Arredondo-Arechavala M., Wang Y.-C., Slater T. J. A., Haigh S. J., Beale A. M., Thompson J. M. (2020). Catal. Sci. Technol..

[cit43] Yevick A., Frenkel A. I. (2010). Phys. Rev. B: Condens. Matter Mater. Phys..

[cit44] Duan Z., Li Y., Timoshenko J., Chill S. T., Anderson R. M., Yancey D. F., Frenkel A. I., Crooks R. M., Henkelman G. (2016). Catal. Sci. Technol..

[cit45] Timoshenko J., Duan Z., Henkelman G., Crooks R. M., Frenkel A. I. (2019). Annu. Rev. Anal. Chem..

[cit46] Becknell N., Son Y., Kim D., Li D., Yu Y., Niu Z., Lei T., Sneed B. T., More K. L., Markovic N. M., Stamenkovic V. R., Yang P. (2017). J. Am. Chem. Soc..

[cit47] Chen S.-A., Liang Y.-C., Lu K.-T., Pao C.-W., Lee J.-F., Lin T.-L., Chen J.-M. (2014). Phys. Chem. Chem. Phys..

[cit48] Frenkel A. I., Wang Q., Sanchez S. I., Small M. W., Nuzzo R. G. (2013). J. Chem. Phys..

[cit49] Becknell N., Kang Y., Chen C., Resasco J., Kornienko N., Guo J., Markovic N. M., Somorjai G. A., Stamenkovic V. R., Yang P. (2015). J. Am. Chem. Soc..

[cit50] van der Hoeven J. E. S., Welling T. A. J., Silva T. A. G., van den Reijen J. E., La Fontaine C., Carrier X., Louis C., van Blaaderen A., de Jongh P. E. (2018). ACS Nano.

[cit51] Huang W.-F., Zhang Q., Zhang D.-F., Zhou J., Si C., Guo L., Chu W.-S., Wu Z.-Y. (2013). J. Phys. Chem. C.

[cit52] Scofield M. E., Zhou Y., Yue S., Wang L., Su D., Tong X., Vukmirovic M. B., Adzic R. R., Wong S. S. (2016). ACS Catal..

[cit53] Yang S., Hong F., Wang L., Guo S., Song X., Ding B., Yang Z. (2010). J. Phys. Chem. C.

[cit54] Liu R., Liu J.-f., Jiang G.-b. (2010). Chem. Commun..

[cit55] Ye F., Liu H., Yang J., Cao H., Yang J. (2013). Dalton Trans..

[cit56] Yu Y., Yang W., Sun X., Zhu W., Li X. Z., Sellmyer D. J., Sun S. (2014). Nano Lett..

[cit57] Zhao W., Huang D., Yuan Q., Wang X. (2016). Nano Res..

[cit58] Zhao W., Ni B., Yuan Q., Wang Y., Zhang Q., Wang X. (2017). Langmuir.

[cit59] Ravel B., Newville M. (2005). J. Synchrotron Radiat..

[cit60] Halder A., Ravishankar N. (2007). Adv. Mater..

[cit61] Mourdikoudis S., Liz-Marzan L. M. (2013). Chem. Mater..

[cit62] Peng Z., You H., Yang H. (2010). ACS Nano.

[cit63] Poudyal N., Chaubey G. S., Nandwana V., Rong C.-b., Yano K., Liu J. P. (2008). Nanotechnology.

[cit64] Yin A.-X., Liu W.-C., Ke J., Zhu W., Gu J., Zhang Y.-W., Yan C.-H. (2012). J. Am. Chem. Soc..

[cit65] Frenkel A. I., Hills C. W., Nuzzo R. G. (2001). J. Phys. Chem. B.

[cit66] Cowley J. M. (1965). Phys. Rev..

[cit67] Lawrence R. L., Olagunju M. O., Liu Y., Mahalingham K., Slocik J. M., Naik R. R., Frenkel A. I., Knecht M. R. (2021). Cat. Sci. Technol..

[cit68] Frenkel A. I., Machavariani V. S., Rubshtein A., Rosenberg Y., Voronel A., Stern E. A. (2000). Phys. Rev. B: Condens. Matter Mater. Phys..

[cit69] Hwang B.-J., Sarma L. S., Chen J.-M., Chen C.-H., Shih S.-C., Wang G.-R., Liu D.-G., Lee J.-F., Tang M.-T. (2005). J. Am. Chem. Soc..

[cit70] Cheng G., Carter J. D., Guo T. (2004). Chem. Phys. Lett..

[cit71] Wang K.-W., Yu Z., Hu A., Hsu Y.-Y., Chen T.-L., Lin C.-Y., Hu C.-W., Yang Y.-T., Chen T.-Y. (2017). RSC Adv..

[cit72] Hume-Rothery W., Mabbott G. W., Evans K. M. C. (1934). Philos. Trans. R. Soc., A.

[cit73] Godfrey I. J., Dent A. J., Parkin I. P., Maenosono S., Sankar G. (2017). J. Phys. Chem. C.

[cit74] Samsonov V. M., Bembel A. G., Kartoshkin A. Y., Vasilyev S. A., Talyzin I. V. (2018). J. Therm. Anal. Calorim..

[cit75] Ferrando R., Jellinek J., Johnston R. L. (2008). Chem. Rev..

[cit76] Li Z. Y., Wilcoxon J. P., Yin F., Chen Y., Palmer R. E., Johnston R. L. (2008). Faraday Discuss..

[cit77] Deng L., Hu W., Deng H., Xiao S., Tang J. (2011). J. Phys. Chem. C.

[cit78] He X., Zhang S.-E., Cheng F., Chen Z.-X. (2018). Chem. Commun..

