# Supplementary material for: Reconciling structure prediction of alloyed, ultrathin nanowires with spectroscopy
Source: Chem Sci. 2021 Apr 26;12(20):7158–73. doi: 10.1039/d1sc00627d (PMC8153242; doi:10.1039/d1sc00627d)
Supplement: SC-012-D1SC00627D-s001 [file SC-012-D1SC00627D-s001.pdf]

Electronic Supplementary Material (ESI) for Chemical Science.
This journal is © The Royal Society of Chemistry 2021

Supporting Information for

Reconciling Structure Prediction of Alloyed, Ultrathin Nanowires with Spectroscopy

Scott C. McGuire,[1] Amani M. Ebrahim,[2] Nathaniel Hurley,[1] Lihua Zhang,[3]

Anatoly I. Frenkel,[2,4,*] and Stanislaus S. Wong[1,*]

[1]Department of Chemistry, Stony Brook University,

Stony Brook, New York 11794-3400, USA

[2]Department of Materials Science and Chemical Engineering,

Stony Brook University, Stony Brook, New York 11794-2275, USA

[3]Center for Functional Nanomaterials,

Brookhaven National Laboratory, Upton, New York 11973, USA

[4]Chemistry Division, Brookhaven National Laboratory, Upton, New York 11973, USA

[*]To whom correspondence should be addressed.

Email: anatoly.frenkel@stonybrook.edu; stanislaus.wong@stonybrook.edu

Importance of Morphology: A recent report has demonstrated the superior catalytic performance of PtRu nanowires, as compared with nanorods and nanocubes of similar composition.[1] The as-prepared nanowires exhibited a mass activity that was 1.21 and 2.28 times greater than what was measured for nanorods and nanocubes, respectively. This significant increase in performance can be attributed to the fact that nanowires expose more (111) facets as compared with nanocubes, which are terminated by (100) facets. In addition, the presence of defects within the 1D nanostructures can also influence their catalytic performance.

Typically, nanowires consist of smooth surfaces which are mostly free of defects. However, wavy nanowires can be synthesized, which can be defect rich. For example, Zhang *et al*. reported on the synthesis of two types of Pd_4Sn nanowires, namely penta-twinned and wavy, and probed the effect of surface defects on electrocatalytic performance.[2] The penta-twinned nanowires exhibited smooth surfaces and no surface defects, whereas the wavy nanowires were defect rich. Methanol oxidation reaction (MOR) and oxygen reduction reaction (ORR) measurements both indicated that the wavy nanowires exhibit a much higher activity for both of these reactions as compared with not only their penta-twinned nanowire counterparts but also both the Pt/C and Pd/C commercial standards. It was postulated that presence of abundant surface defects changed the electronic structure of the metals and increased the content of Pd^0 and Sn^{x+}, which could thereby allow for more opportunities for the adsorption, activation, and dissociation of small molecules, thereby leading to an increase in catalytic performance.

Importance of Chemical Composition: Whereas the formation of Pt-based bimetallic alloys certainly improves CO tolerance and catalytic activity, additional enhancements can be achieved by the generation of more complex ternary alloy systems, such as PtRuM, wherein M is a transition metal. The production of ternary alloys can further promote the generation of adsorbed

oxygen-containing species, such as OH, which can thereby lead to the oxidation of CO, while simultaneously reducing the cost of the catalysts.

As an example, our group has demonstrated that PtRuFe alloy nanowires yield specific activities which are 2 and 11 times greater than those achieved for PtRu and PtFe nanowires, respectively.[3] Furthermore, the MOR performance could be additionally tuned by controlling the chemical composition within the PtRuFe nanowires; in effect, nanowires possessing a composition of Pt_7Ru_2Fe yielded the highest activities.

Other groups have also performed a number of relevant studies. For example, PtRuCu hexapods have been reported to exhibit specific activities which are around two times greater than those measured for PtCu/C.[4] PtRuM-O (M = Ni, Fe, or Co) nanowires have also been studied to determine the effect of the transition metal within the alloy.[5] In effect, $Pt_{62}Ru_{18}Ni_{20}$-O, $Pt_{61}Ru_{16}Fe_{23}$-O, and $Pt_{59}Ru_{19}Co_{22}$-O nanowires have achieved mass activities of 2.72, 2.15 and 2.06 A mg_{pt}^{-1}, respectively, which are much greater than what was measured for $Pt_{65}Ru_{35}$ nanowires (0.46 A mg_{pt}^{-1}) alone. It has also been demonstrated that the synthesis of more complicated quaternary nanoscale alloys can further improve catalytic performance.[6, 7] Overall, alloy formation can be used to tune the binding energies, *d*-band centers, and lattice parameters, all of which can lead to significant beneficial impacts upon electrocatalytic performance.

Examples of RuCo for Electrocatalysis: RuCo alloys embedded within N-doped carbon have been shown to exhibit impressive HER catalytic activities within alkaline, acidic, and neutral media.[8] Specifically, the RuCo electrocatalysts can realize a current density of 10 mA cm^{-2} for HER at low overpotentials of 34, 6, and 60 mV within 1.0 M KOH, 0.5 M $HClO_4$, and 1.0 M phosphate buffered saline (PBS) solutions, respectively, all of which are lower values than those achieved using the Pt/C standard alone. In a separate study, CoRu nanoparticles supported onto

carbon quantum dots (CoRu/CQDs) were also shown to give rise to excellent performance for HER, wherein the activity could be tuned by controlling the composition of the CoRu nanoparticles.[9] $CoRu_x$ (x = 0.1, 0.3, 0.5, 0.7, 1.0) samples, where 'x' indicates the Ru/Co ratio, were prepared and measured for HER, which showed that the $CoRu_{0.5}$ sample yielded the highest activity out of the $CoRu_x$ alloys and outperformed the Pt/C standard. The increase in activity observed in the $CoRu_x$/CQDs electrocatalysts was determined to be caused by the lattice strain as a result of alloying and electronic coupling between Co and Ru.

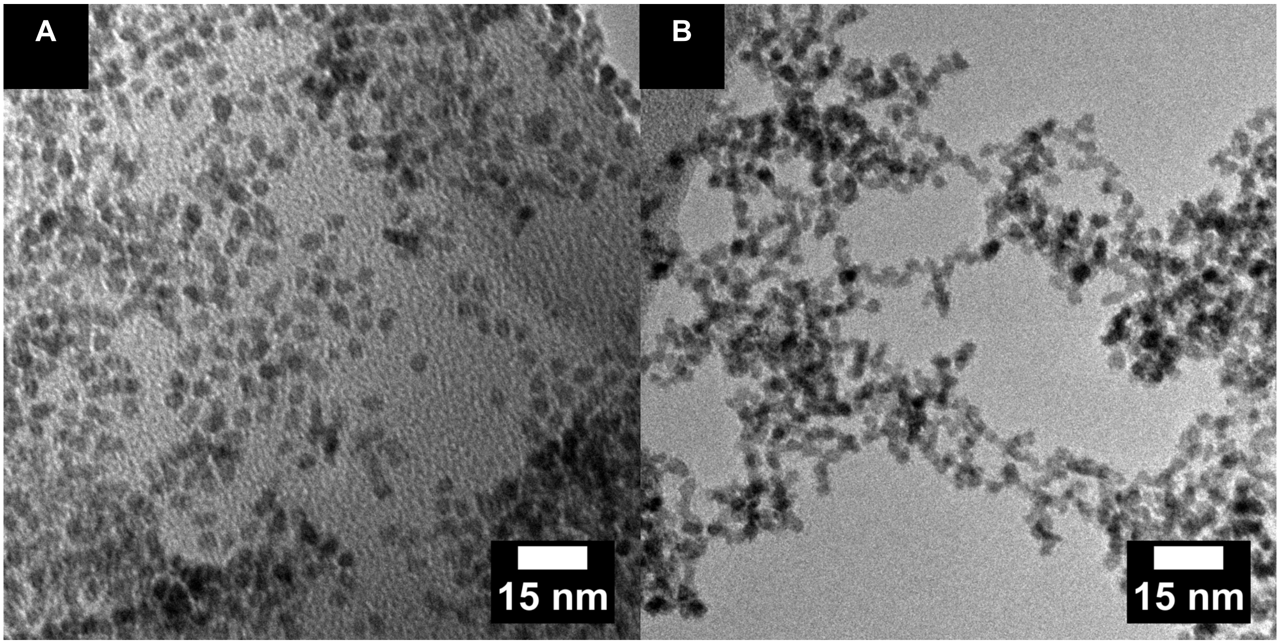

Figure S1. TEM images of (A) Ru NPs synthesized using only OAm and of (B) Ru NWs synthesized using OAm and OAc.

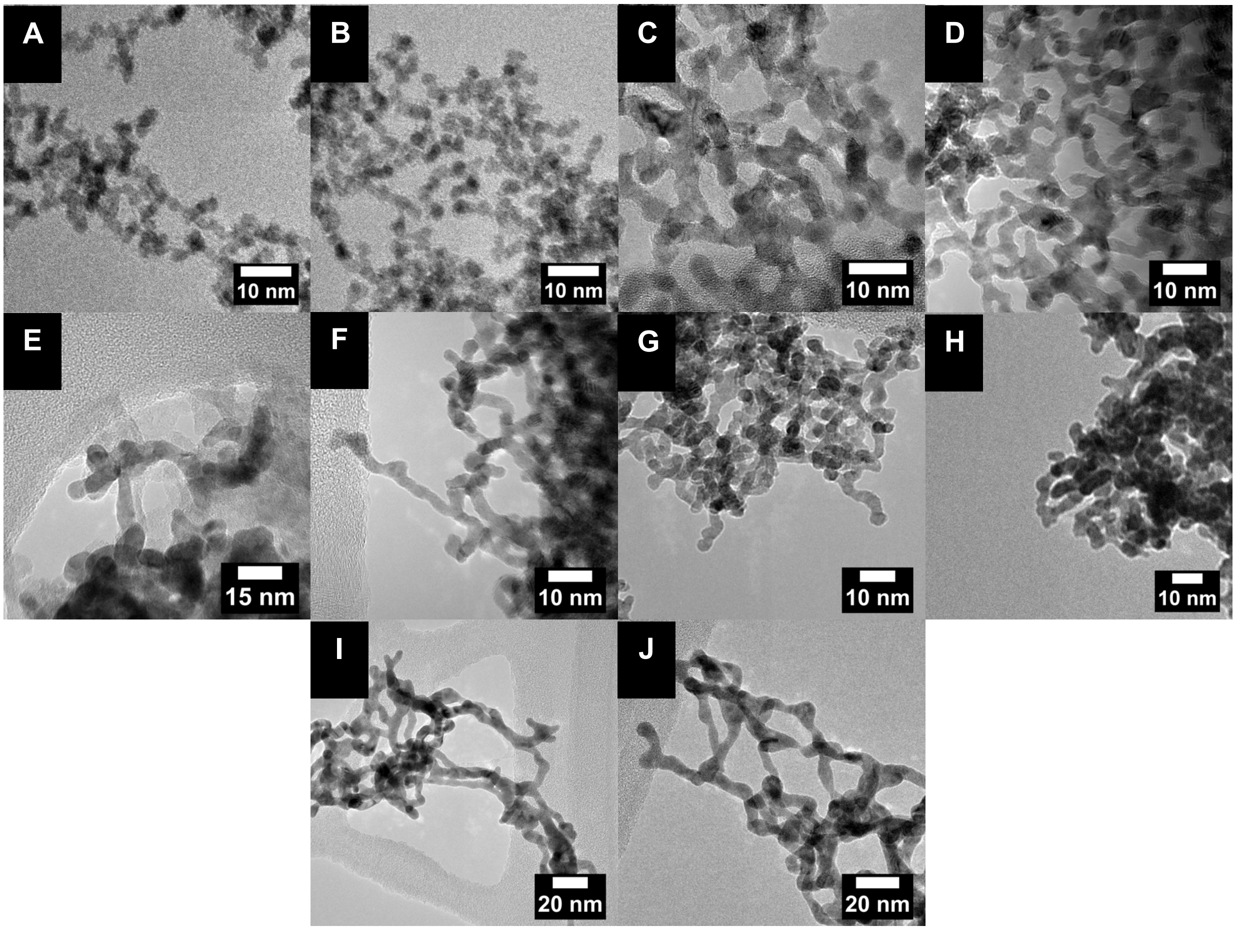

Figure S2. TEM images corresponding to (A) Ru-S, (B) Ru_2Co_1, (C) Ru-H, (D) Ru_1Pt_1, (E) Pd, (F) Pt, (G) Pd_1Pt_1, (H) Pd_1Pt_9, (I) Au, and (J) Au_1Ag_1 NWs, respectively.

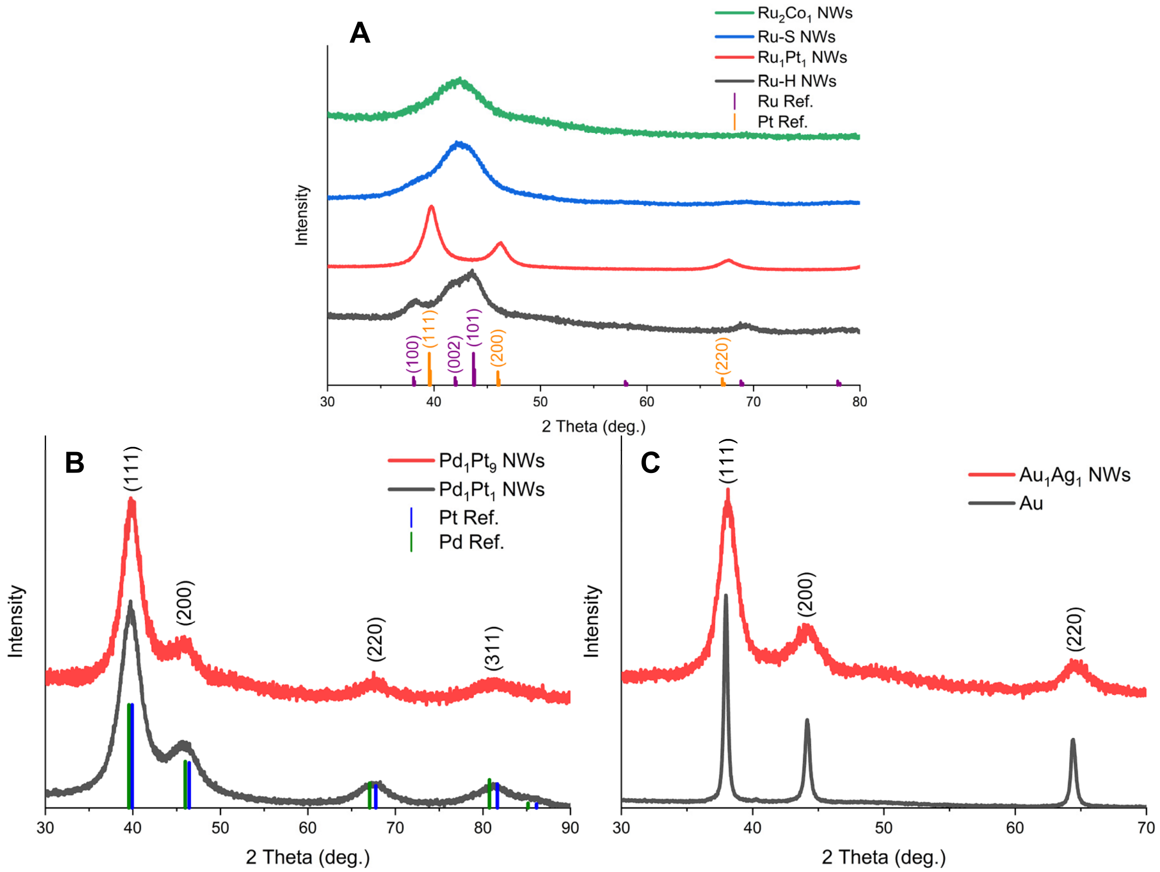

Figure S3. XRD patterns corresponding to (A) Ru-based NWs, (B) PdPt NWs, and (C) Au and Au_1Ag_1 NWs.

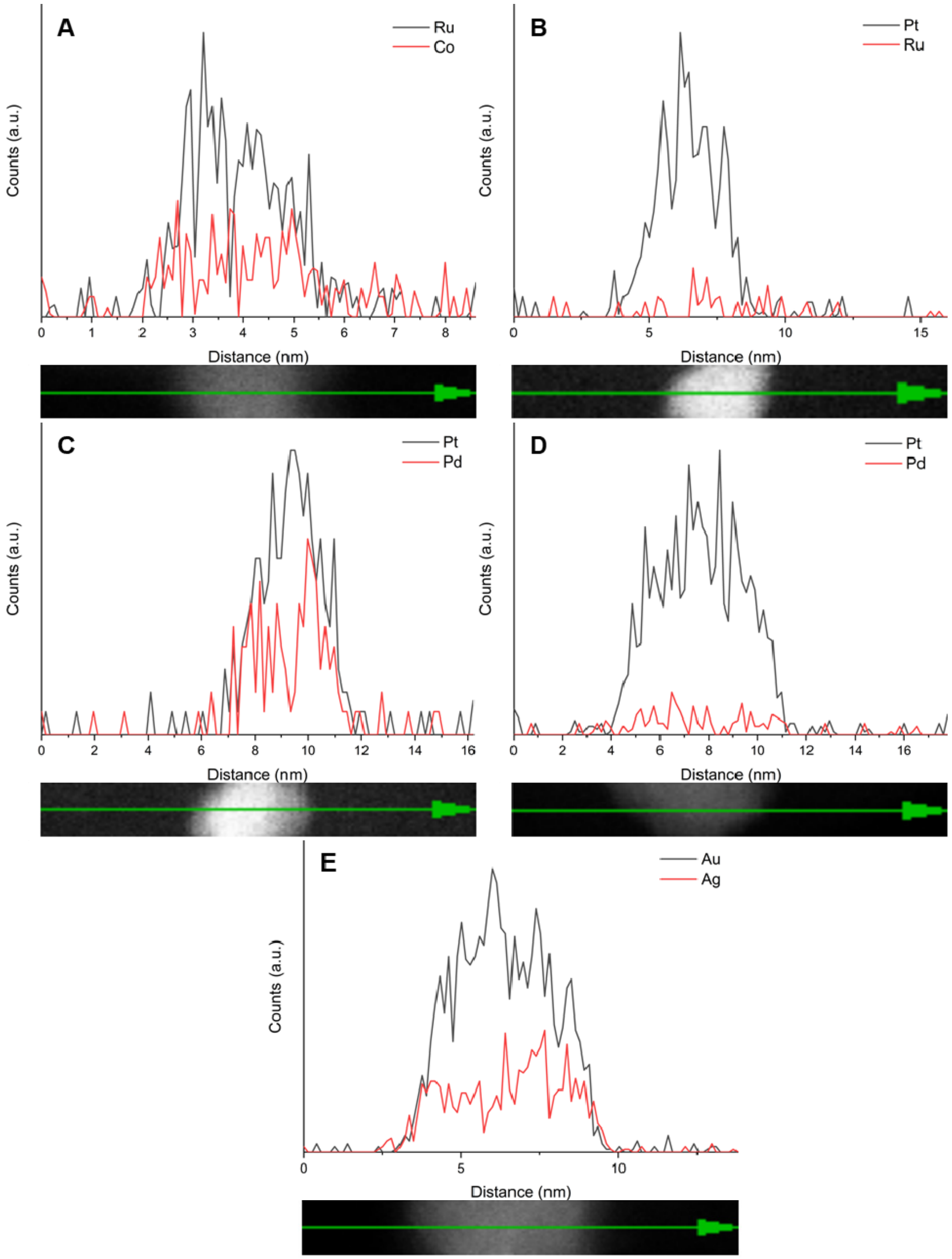

Figure S4. EDS line-scan spectra taken along the solid green lines shown (from left to right) with corresponding HAADF-STEM images (beneath) for representative NWs of (A) Ru_2Co_1, (B) Ru_1Pt_1, (C) Pd_1Pt_1, (D) Pd_1Pt_9, and (E) Au_1Ag_1 analyzed in this study.

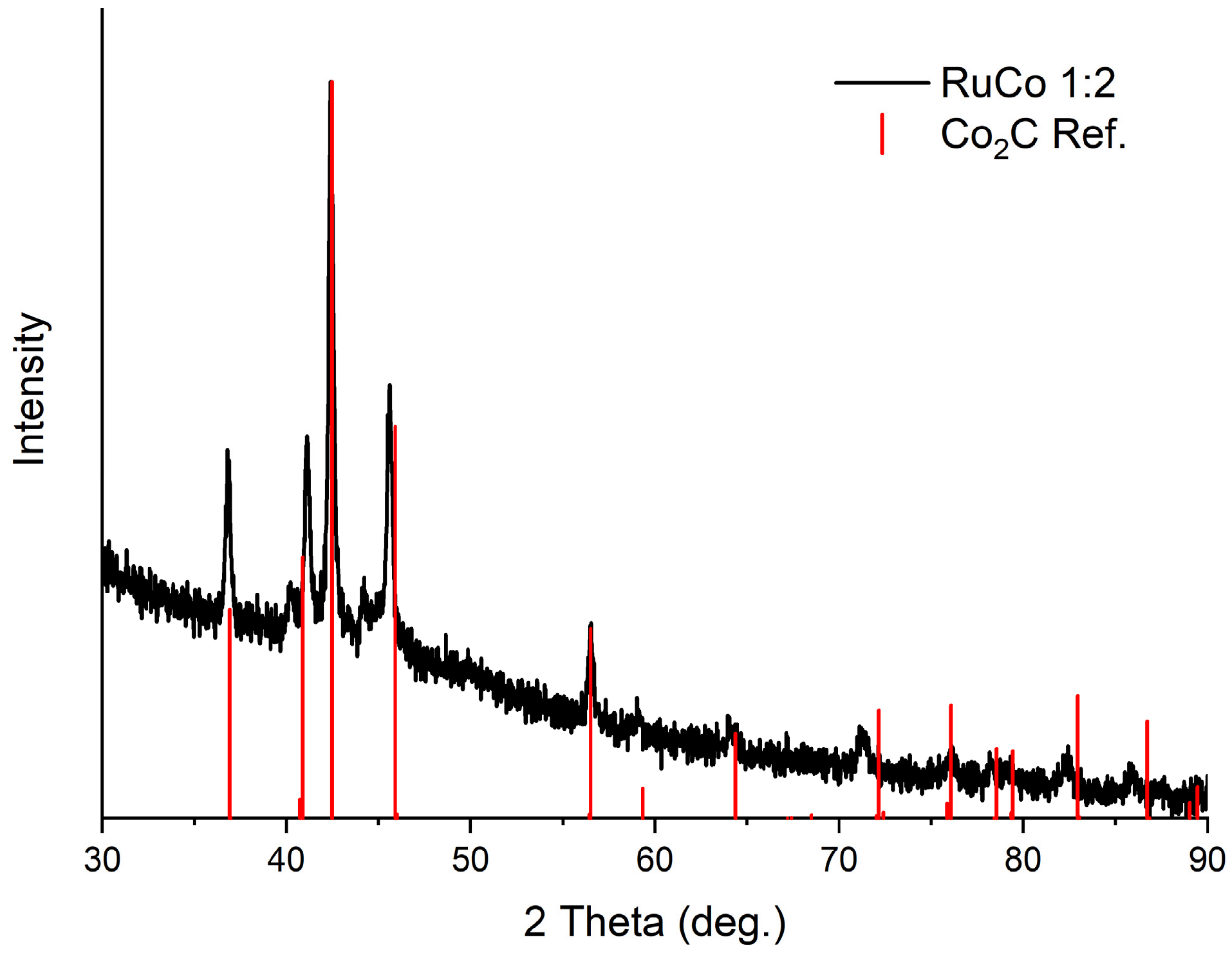

Figure S5. XRD pattern of RuCo NWs, synthesized using a Ru: Co feed ratio of 1: 2. The reference pattern for Co_2C is shown in red.

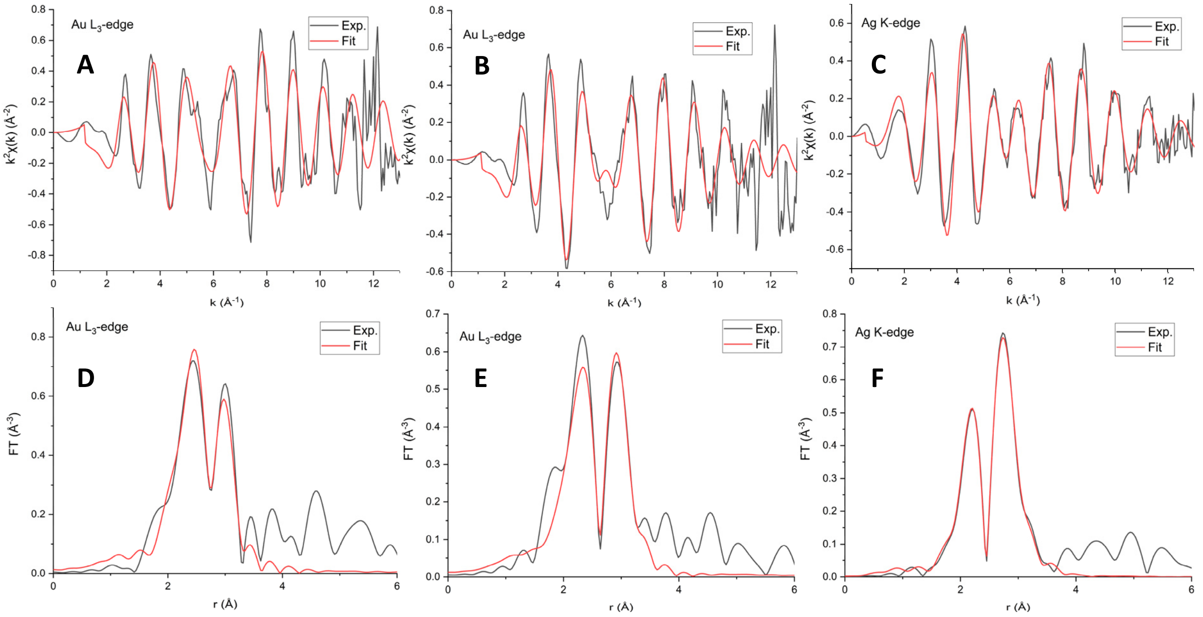

Figure S6. EXAFS fitting results for (A, D) Au NWs and (B, C, E, F) Au_1Ag_1 NWs in (A-C) k-space and (D-F) r-space, respectively.

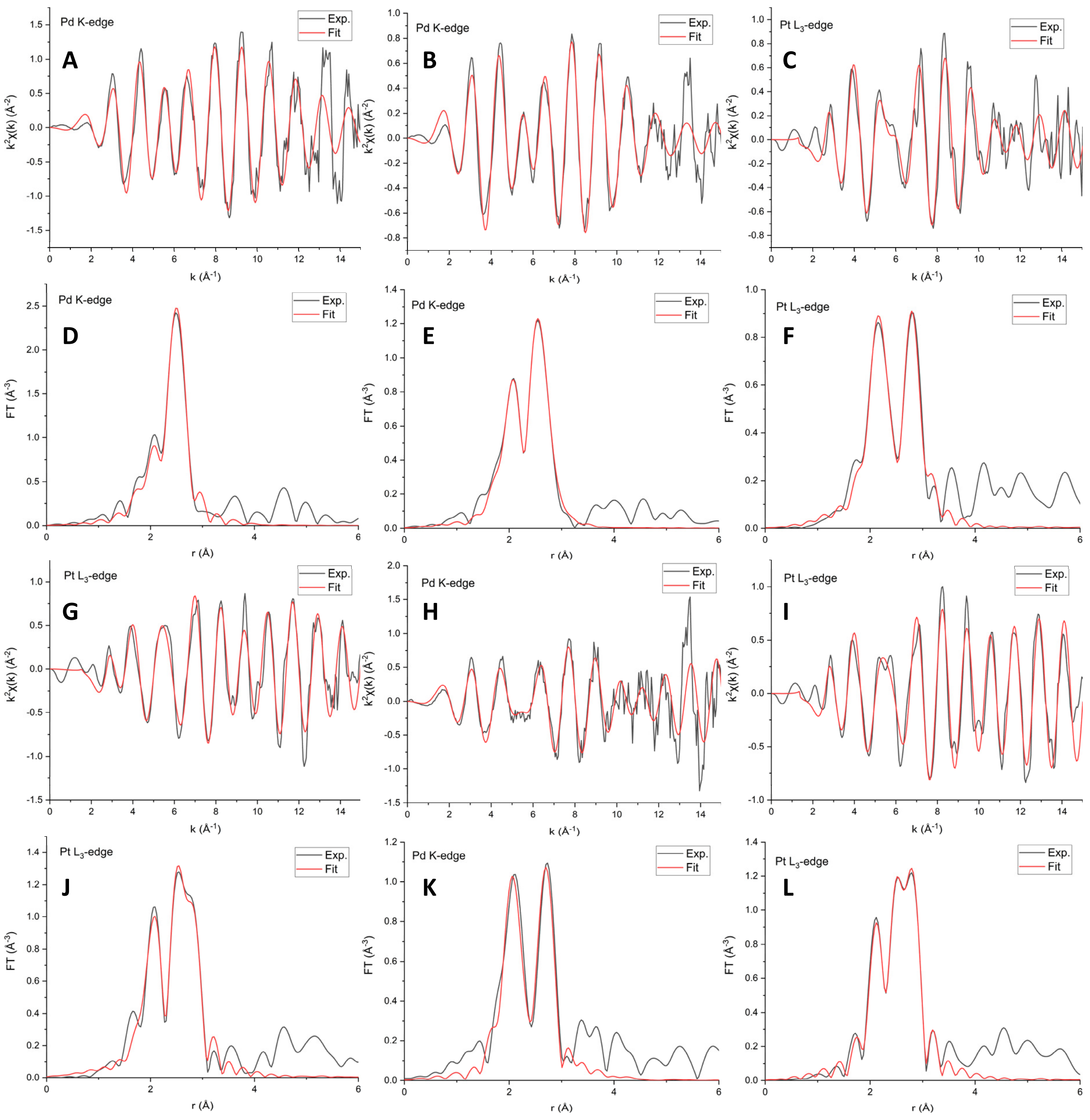

Figure S7. EXAFS fitting results for (A, D) Pd NWs, (B, C, E, F) Pd_1Pt_1 NWs, (G, J) Pt NWs, and (H, I, K, L) Pd_1Pt_9 NWs in (A-C, G-I) k-space and (D-F, J-L) r-space, respectively.

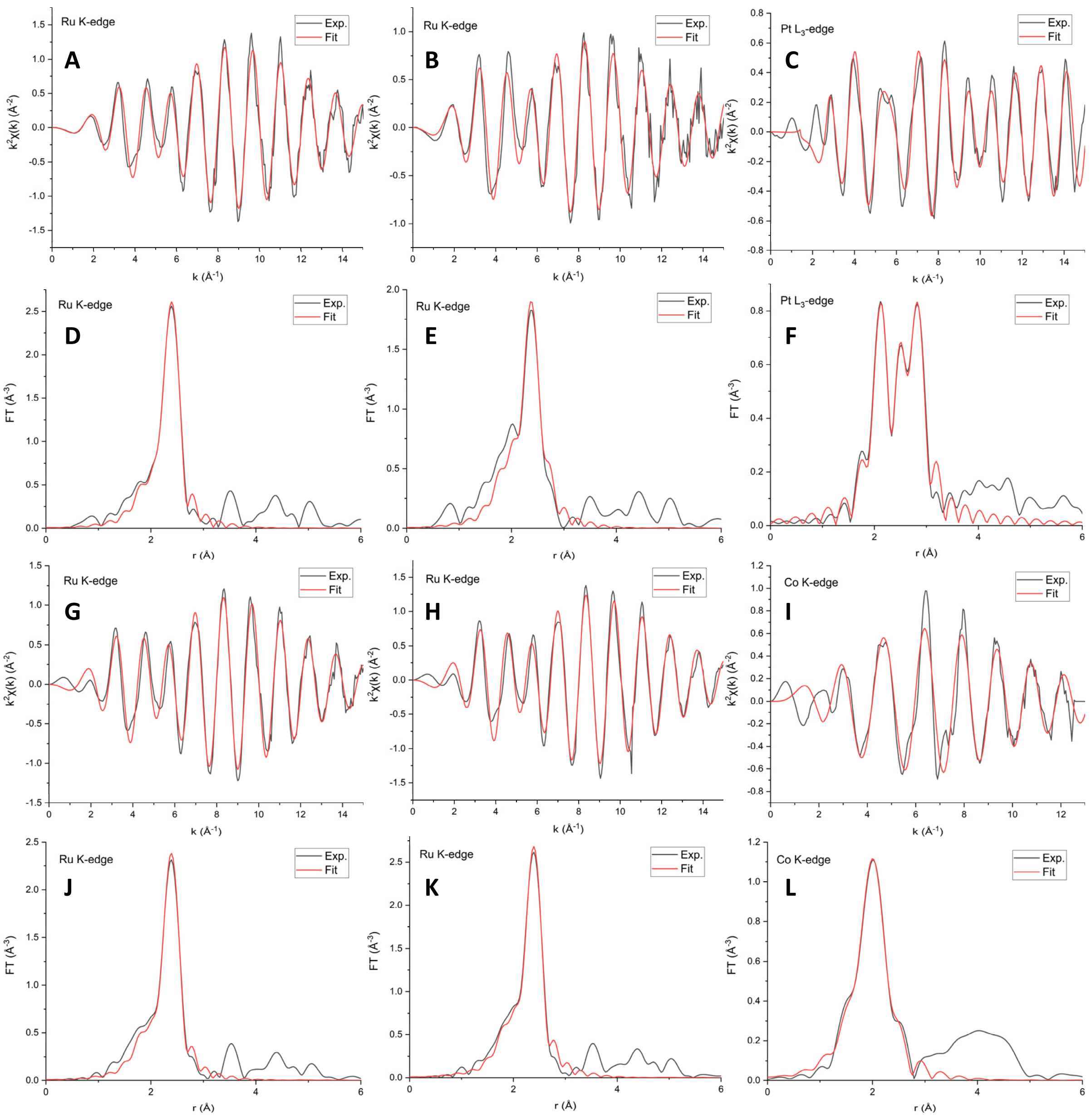

Figure S8. EXAFS fitting results corresponding to (A, D) Ru-H NWs, (B, C, E, F) Ru_1Pt_1 NWs, (G, J) Ru-S NWs, and (H, I, K, L) Ru_2Co_1 NWs in (A-C, G-I) k-space and (D-F, J-L) r-space, respectively.

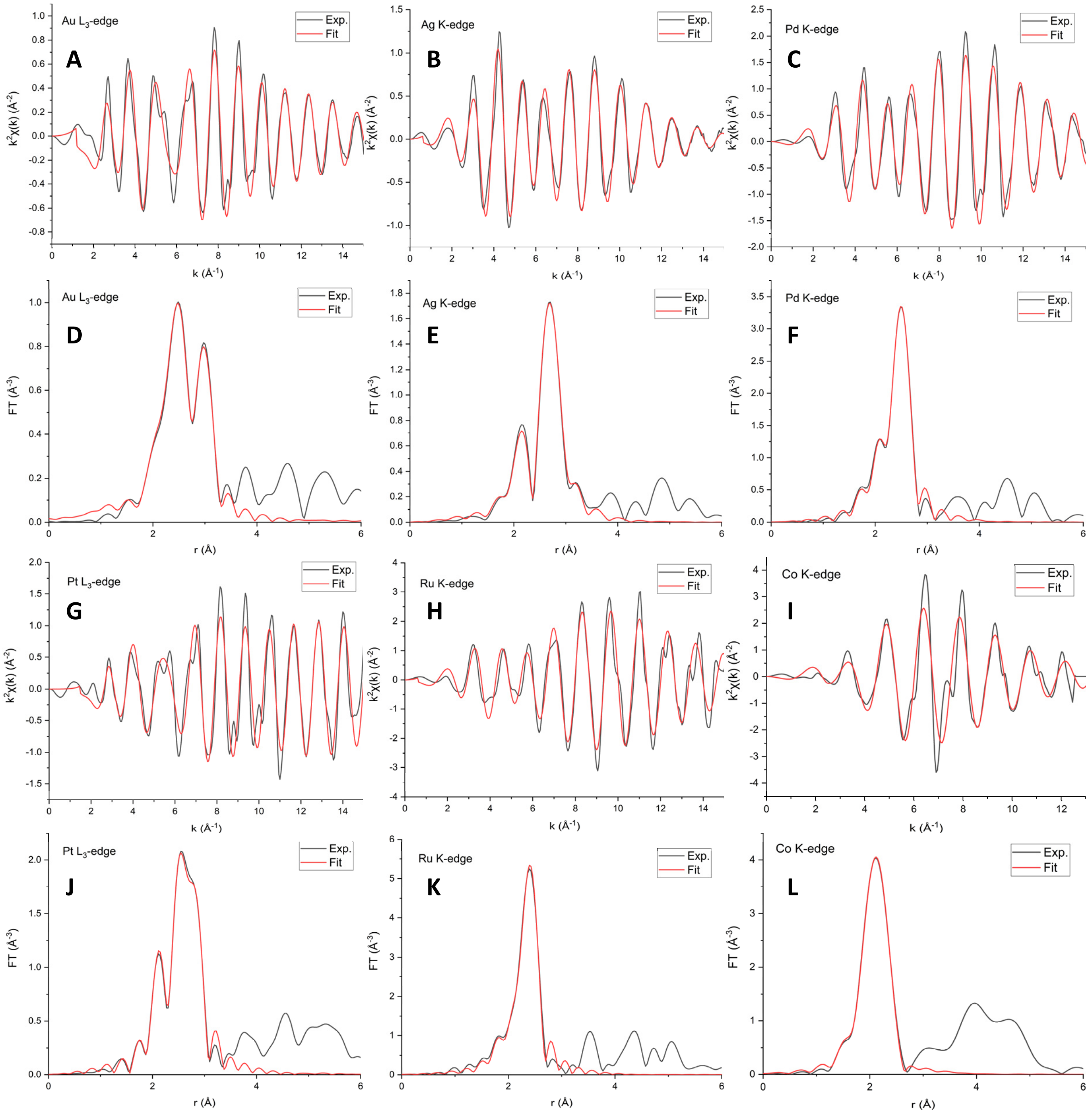

Figure S9. EXAFS fitting results in (A-B, G-I) k-space and (D-F, J-L) r-space for the (A, D) Au, (B, E) Ag, (C, F) Pd, (G, J) Pt, (H, K) Ru, and (I, L) Co foils, respectively.

Table S1. Structural parameters of Au and Au_1Ag_1 NWs, as derived from EXAFS analysis.

Sample	Contribution	N	R (Å)	σ^2 (Å^2)
Au Foil	Au-Au	12	2.862 ± 0.003	0.0081 ± 0.0004
Ag Foil	Ag-Ag	12	2.865 ± 0.003	0.0097 ± 0.0004
Au NWs	Au-Au	10.3 ± 1.2	2.857 ± 0.007	0.009 ± 0.001
Au_1Ag_1 NWs	Au-Au	7.4 ± 0.9	2.84 ± 0.01	0.010 ± 0.001
	Au-Ag	2.9 ± 0.9	2.86 ± 0.01	0.011 ± 0.002
	Ag-Ag	4.1 ± 0.5	2.864 ± 0.008	0.008 ± 0.001
	Ag-Au	4.7 ± 0.9	2.86 ± 0.01	0.011 ± 0.002

Table S2. Structural parameters of Pd, Pt, and PdPt NWs, as derived from EXAFS analysis.

Sample	Contribution	N	R (Å)	σ^2 (Å^2)
Pd Foil	Pd-Pd	12	2.740 ± 0.002	0.0054 ± 0.0003
Pt Foil	Pt-Pt	12	2.765 ± 0.002	0.0048 ± 0.0002
Pt NWs	Pt-Pt	7.6 ± 1.1	2.762 ± 0.005	0.0049 ± 0.0007
Pd NWs	Pd-Pd	10.6 ± 0.8	2.744 ± 0.003	0.0066 ± 0.0005
Pd_1Pt_1 NWs	Pd-Pd	5.4 ± 0.5	2.742 ± 0.004	0.0054 ± 0.0006
	Pd-Pt	4.0 ± 0.5	2.740 ± 0.005	0.0050 ± 0.0007
	Pt-Pt	6.3 ± 0.6	2.738 ± 0.004	0.0055 ± 0.0006
	Pt-Pd	2.9 ± 0.4	2.740 ± 0.005	0.0050 ± 0.0007
Pd_1Pt_9 NWs	Pd-Pd	1.4 ± 1.1	2.73 ± 0.02	0.000 ± 0.004
	Pd-Pt	7.6 ± 1.9	2.740 ± 0.01	0.004 ± 0.002
	Pt-Pt	8.2 ± 0.4	2.750 ± 0.003	0.0046 ± 0.0002
	Pt-Pd	0.8 ± 0.2	2.740 ± 0.01	0.004 ± 0.002

Table S3. Structural parameters of Ru and Ru_1Pt_1 NWs, synthesized by the hydrothermal method, as derived from EXAFS analysis.

Sample	Contribution	N	R (Å)	σ^2 (Å^2)
Ru Foil	Ru-Ru	12	2.676 ± 0.002	0.0040 ± 0.0003
Pt Foil	Pt-Pt	12	2.765 ± 0.002	0.0048 ± 0.0002
Ru-H NWs	Ru-Ru	6.9 ± 0.4	2.672 ± 0.003	0.0049 ± 0.0003
Ru_1Pt_1 NWs	Ru-Ru	6.8 ± 1.1	2.671 ± 0.006	0.0060 ± 0.0009
	Ru-Pt	1.1 ± 0.6	2.697 ± 0.009	0.003 ± 0.002
	Pt-Pt	7.8 ± 0.5	2.740 ± 0.004	0.0064 ± 0.0004
	Pt-Ru	0.9 ± 0.3	2.697 ± 0.009	0.003 ± 0.002

Table S4. Structural parameters of Ru and Ru_2Co_1 NWs, synthesized by the OAm/OAc method, as derived from EXAFS analysis.

Sample	Contribution	N	R (Å)	σ^2 (Å^2)
Ru Foil	Ru-Ru	12	2.676 ± 0.002	0.0040 ± 0.0003
Co Foil	Co-Co	12	2.494 ± 0.002	0.0065 ± 0.0002
Ru-S NWs	Ru-Ru	7.3 ± 0.5	2.667 ± 0.003	0.0058 ± 0.0004
Ru_2Co_1 NWs	Ru-Ru	8.5 ± 0.7	2.662 ± 0.004	0.0058 ± 0.0004
	Ru-Co	0.4 ± 0.4	2.608 ± 0.008	0.005 ± 0.002
	Co-Co	7.7 ± 1.9	2.50 ± 0.01	0.016 ± 0.003
	Co-Ru	1.8 ± 0.5	2.608 ± 0.008	0.005 ± 0.002

References

1. L. Huang, X. Zhang, Q. Wang, Y. Han, Y. Fang and S. Dong, *J. Am. Chem. Soc.*, 2018, **140**, 1142-1147.
2. Y. Zhang, B. Huang, Q. Shao, Y. Feng, L. Xiong, Y. Peng and X. Huang, *Nano Lett.*, 2019, **19**, 6894-6903.
3. M. E. Scofield, C. Koenigsmann, L. Wang, H. Liu and S. S. Wong, *Energy Environ. Sci.*, 2015, **8**, 350-363.
4. S. Xue, W. Deng, F. Yang, J. Yang, I. S. Amiinu, D. He, H. Tang and S. Mu, *ACS Catal.*, 2018, **8**, 7578-7584.
5. H. Li, Y. Pan, D. Zhang, Y. Han, Z. Wang, Y. Qin, S. Lin, X. Wu, H. Zhao, J. Lai, B. Huang and L. Wang, *J. Mater. Chem. A*, 2020, **8**, 2323-2330.
6. Q.-X. Chen, C.-X. Yu, H.-H. Li, Z. He and J.-W. Liu, *Inorg. Chem.*, 2020, **59**, 1376-1382.
7. C. Yao, H. Xu, A. Li, J. Li, F. Pang, P. Zhao, J. He, W. Yi, Y. Jiang and L. Huang, *RSC Adv.*, 2020, **10**, 3579-3587.
8. F. Zhang, Y. Zhu, Y. Chen, Y. Lu, Q. Lin, L. Zhang, S. Tao, X. Zhang and H. Wang, *J. Mater. Chem. A*, 2020, **8**, 12810-12820.
9. W. Li, Y. Zhao, Y. Liu, M. Sun, G. I. N. Waterhouse, B. Huang, K. Zhang, T. Zhang and S. Lu, *Angew. Chem., Int. Ed.*, 2020, DOI: 10.1002/anie.202013985, Ahead of Print.
